# Novel variants in Krueppel like factor 1 that cause persistence of fetal hemoglobin in In(Lu) individuals

**DOI:** 10.1038/s41598-021-97149-y

**Published:** 2021-09-17

**Authors:** Jesse Eernstman, Barbera Veldhuisen, Peter Ligthart, Marieke von Lindern, C. Ellen van der Schoot, Emile van den Akker

**Affiliations:** 1 Sanquin Research, Department of Hematopoiesis, Amsterdam, The Netherlands, and Landsteiner Laboratory, Amsterdam UMC, University of Amsterdam, Amsterdam, The Netherlands; 2 Sanquin Research, department of Immunohematology Experimental, Amsterdam, The Netherlands, and Landsteiner Laboratory, Amsterdam UMC, University of Amsterdam, Amsterdam, The Netherlands; 3 Department of Immunohematology Experimental, Sanquin Research, Amsterdam, The Netherlands

**Keywords:** Medical research, Haematological diseases, Haematopoietic stem cells

## Abstract

Beta-hemoglobinopathies become prominent after birth due to a switch from γ-globin to the mutated β-globin. Haploinsufficiency for the erythroid specific indispensable transcription factor Krueppel-like factor 1 (KLF1) is associated with high persistence of fetal hemoglobin (HPFH). The In(Lu) phenotype, characterized by low to undetectable Lutheran blood group expression is caused by mutations within *KLF1* gene. Here we screened a blood donor cohort of 55 Lutheran weak or negative donors for KLF1 variants and evaluated their effect on KLF1 target gene expression. To discriminate between weak and negative Lutheran expression, a flow cytometry (FCM) assay was developed to detect Lu antigen expression. The Lu(a−b−) (negative) donor group, showing a significant decreased CD44 (Indian blood group) expression, also showed increased HbF and HbA2 levels, with one individual expressing HbF as high as 5%. *KLF1* exons and promoter sequencing revealed variants in 80% of the Lutheran negative donors. Thirteen different variants plus one high frequency SNP (c.304 T > C) were identified of which 6 were novel. In primary erythroblasts, knockdown of endogenous KLF1 resulted in decreased CD44, Lu and increased HbF expression, while KLF1 over-expressing cells were comparable to wild type (WT). In line with the pleiotropic effects of KLF1 during erythropoiesis, distinct KLF1 mutants expressed in erythroblasts display different abilities to rescue CD44 and Lu expression and/or to affect fetal (HbF) or adult (HbA) hemoglobin expression. With this study we identified novel KLF1 variants to be include into blood group typing analysis. In addition, we provide further insights into the regulation of genes by KLF1.

## Introduction

Blood group phenotyping of donors is crucial to provide matched blood to anemic patients to prevent transfusion reactions and alloimmunization. The genetic basis of most blood groups have been elucidated allowing for genetic prediction of blood group phenotype through detection of prevalent small nucleotide polymorphism (SNP), insertion or deletions. Importantly, whole genome sequencing, amplicon or PCR based genetic screenings may identify specific novel variants of which the phenotypic translation may be difficult to predict. Therefore, the identification of novel genetic variants that lead to blood group null and weak phenotypes is still important to supplement existing high throughput genotyping. During erythropoiesis the indispensable erythroid specific transcription factor Krueppel like factor 1 (KLF1, formerly erythroid KLF) transcriptionally regulates expression of specific blood group antigens. KLF1 variants can cause a multitude of phenotypes. These vary from benign loss of specific blood groups to inherently dominant diseases like congenital dyserythropoiesis type IV (CDA IV). *KLF1* variants have been divided into different classes (class 1 to 4) according to their location within (and effect on) the gene^1^. One of the blood group systems affected by KLF1 variants is Lutheran (Lu), encoded by the *BCAM* gene^2^. BCAM is a glycoprotein containing 25 Lu antigens and putatively functions as a laminin binding receptor^3^. BCAM negativity is rare (1:3000; 0.005–0.032%^4^). It is caused by *KLF1* or *GATA1* mutations, but also deletions/stop variants in the *BCAM* gene itself causes negativity for BCAM^2,5,6^. *KLF1* variants specifically result in the dominant type of Lu(a−b−), termed In(Lu) *or* inhibitor of Lu. Recessive Lu(a−b−), also known as the [Lu_null_ phenotype], is caused by variants in the *BCAM* gene. This results in complete absence of Lu on blood and other tissue and may increase the risk of alloimmunization upon unmatched transfusions. Clinically, antibodies of the Lutheran system are relatively benign. Lutheran antibodies have not been implicated in immediate haemolytic transfusion reactions (HTR)^7^. However, mild delayed hemolytic transfusion reactions (DHTR) can be caused by anti-Lu(a) and anti-Lu(b)^8^. Anti-Lu3 (previously known as anti-Lu^ab^) are rare antibodies found in alloimmunised individuals with the recessive type of Lu(a–b–) ([Lu_null_ phenotype], caused by variants in the *BCAM* gene). These individuals should only receive Lu(a − b −). The mixed field nature of Lu agglutination reactions makes proper assessment of Lu weak and negative individuals difficult^9^. KLF1 also regulates specific beta-locus globin expression, either directly or through the actions of target genes encoding transcriptional regulators or chromatin remodelers like LRF and BCL11A^10,11^. KLF1 variants can cause a multitude of phenotypes, some cause benign loss of specific blood groups while others lead to HPFH or the inherently dominant disease, congenital dyserythropoiesis type IV (CDA IV). Class 2 and 3 compound heterozygotes variants can display very high HbF levels of up to 40% of total hemoglobin (Hb). Interestingly, so far it has been reported that both carriers of class 2 or 3 KLF1 variants are able to display the In(Lu) and/or HPFH phenotype^1^. To investigate if HPFH is a common feature of In(Lu) individuals, or whether this is determined by specific variants and to identify novel variants in KLF1, a cohort of In(Lu) donors was investigated. We identified 55 Lu weak or negative individuals of which 80% harbored KLF1 variants with 6 novel variants identified. Lu negative donors with KLF1 variants displayed low levels of CD44 and in some cases, increased HbF (α2γ2), HbA2 (α2δ2) and lower HbA1 levels. These effects on CD44, Hemoglobin Beta (*HBB)* and BCAM RNA expression on in vitro cultured In(Lu) erythroblast have been described before^2^ as the result of decreased binding to the promoters of these genes^12^. However, this was never shown on protein level nor upon ectopic expression of KLF1 mutants, which we now show here. The data indicates that the pleiotropic effects on globin and RBC membrane protein expression are KLF1-variant class specific.

## Materials and methods

### Chemicals

All chemicals were purchased from Sigma-Aldrich (Munich, Germany), the culture reagents from Thermo Fisher Scientific (Waltham, Massachusetts, USA) and growth factors from Peprotech (Pittsburgh, USA) unless otherwise specified.

### Donor blood sample collection and typing

Peripheral blood mononuclear cells (PBMCs) were obtained according to the Declaration of Helsinki (seventh revision, 2013). Written informed consent to extract and use human material (peripheral blood) as part of regular donations was obtained with approval of the local medical ethics committee (MEC; guidelines of NetCord FACT, by the Sanquin Blood bank, The Netherlands). Experimental protocols were approved by Sanquin (MEC). Lutheran blood typing was performed by serology using anti Lu(a) or Lu(b) antibodies (Pelicluster^®^, Sanquin, the Netherlands) in standard agglutination assays.

### Donor sequencing

Blood collected from Lu negative and weak donors (Supplementary Table 1) and KLF1 exons 1–3 and promoter (Supplementary Table 2), were amplified by PCR cleaned by Exo-SAP^®^ as described by manufacturers (GE Healthcare, Exo Pro Star kit) and sequenced on ABI 3730xl DNA Analyzer (Thermo Fisher).

### Flow cytometry

Erythrocytes were diluted to 1 × 10^8^ cells/ml and incubated with primary antibody (Supplementary Table 3) for 30’ at RT and washed five times with PBS supplemented with 0.5% bovine serum albumin (BSA). Unconjugated antibodies were additionally stained with a secondary antibody goat anti-human APC and incubated for 30’ on ice. Cells were washed once with ice-cold PBS/0.5% BSA. For intracellular staining of globin subunits, cells were fixed using 0.025% glutaraldehyde for 10 min, pelleted (1800 rpm, 5 min) and permeabilized using 0.05% NP40 for 10 min. After pelleting and washing using ice-cold PBS, cells were stained using HbA-PE (Santa Cruz) and HbF-APC (Invitrogen) for 30 min at 4 degrees Celsius. Samples were measured using a Flow Cytometer Canto II (BD Biosciences) and analysed using Flowjo software (Flowjo, LLC, USA).

### HPLC

Hb isoform expression was determined on a minimum of 1 × 10^7^ cells by high-performance cation-exchange liquid chromatography (HPLC) on Waters Alliance 2690 equipment as previously described^13^.

### Blood-MLPA assay

DNA samples from 55 Caucasian donors were extracted and genotyped by Multiplex ligation-dependent probe amplification (MLPA). These donors were genotyped with exon-specific probes for the Lutheran gene to evaluate the relative copy number of each DNA sequence. Each probe was composed of two half-probes (5′ and 3′ half-probes), consisting of a target-specific sequence and a universal primer sequence allowing the simultaneous multiplex PCR amplification of all targets^14^.

### Cloning of novel KLF1 mutants

The ORF from human KLF1 was amplified by PCR using primers with specific restriction enzymes within the oligo’s and cloned into the lentiviral vector PAD5 (pRRLSIN.PPT.hCMV.2A-GFPpre^15^), which was cut using XbaI (NEB, R0145S) and EcoRI (NEB, R0101S). The novel KLF1 variants identified were introduced into the wt KLF1 within PAD5 using Phusion Site-Directed Mutagenesis Kit according to manufacturer’s guidelines (Thermo Fisher; primers in Supplementary Table 4).

### Lentiviral virus production

Human Embryonic Kidney (HEK) 293 T cells (1 × 10^6^ / mL) were cultured within T175 flasks (Sigma Aldrich) in DMEM (Thermo Fisher) supplemented with 10% FCS. Cells were transfected at 50% confluence using 5 μg Env plasmid (pMD2.VSVG), 15 μg of packaging plasmid (pCMVΔR8.91), 20 μg of gateway shRNA lentiviral plasmid or PAD5 containing (mutant) KLF1 in 250 μL water and 250 μL CaCl_2_. Dropwise 500μL HEPES 2 × was added under shaking conditions. After 2 days medium was harvested, spun down to remove cell debris (1800 rpm, RT) and filtered using a 0.45 μm filter. Filtered medium was ultra-centrifuged for 3 h at 20.000 g and resuspended in IMDM medium in 500 μL aliquots, snap frozen with liquid nitrogen and stored at − 80 C until used.

### Primary erythroid cell culturing and transduction with KLF1 constructs

Erythroblasts were cultured from CD34 + cells purified from mobilized peripheral blood (MPB; consent was given in accordance with the declaration of Helsinki and the Sanquin internal ethics board). On day 1 isolated CD34 + we resuspended in Cellquin^16^, supplemented with stem cell factor (SCF, 239 T culture supernatant; 100 ng/ml), TPO (50 ng/nl), IL3 (10 ng/ml), IL6 (10 ng/ml), at a cell density of 1 × 10^6^ cells/mL as described previously^16^. Cells were cultured for 4 days (37 °C, 5% CO2) and medium was refreshed every other day. On day 4, cells were washed and cultured as described previously^16^. On day 7, CD71 + CD235low erythroblasts were transduced with lentivirus containing scrambled or a short hairpin targeting 3’ untranslated region (UTR) of KLF1 mRNA, allowing knock down of endogenous KFL1. 24 h post transduction, shRNA expressing cells were selected by 1 μg/mL puromycin for 48 h. After 48 h the CD71 + CD235low cells were transduced with mutant or wild type KLF1 and directly transferred to erythroid differentiation^16^.

### SDS PAGE and western blotting

Cells were lysed in ice-cold lysis buffer as previously described^17^. Lysates were cleared by centrifugation (10.000 rpm, 4 °C), supplemented with 1 volume of Laemmli buffer and heated for 3 min at 95 °C and run on 24-well pre-cast gels (Biorad). Blotting was performed using iBlot 2 (Life technologies). Membranes were stained with goat anti-human KLF1 antibody (Abcam, clone ab2483) followed by secondary rabbit anti-goat labelled with Horseradish Peroxidase (DAKO, clone p0449), see Supplementary Table 3.

### Statistical analysis

All measurements represent the average MFI of at least three single individual measurements (*** indicates a P value of < 0.001, Tukey's multiple comparisons test or unpaired student T-test as indicated in the figure legends). MFI is indicated as the Mean geometric Fluorescence Intensity of the signal. Statistical analysis was done using GraphPad Prism (version 6.01). First, One Way Anova test was performed, followed by a Tukey's multiple comparisons test.

## Results

### Defining negativity for the mixed field Lu blood group by flow cytometry

**Figure 1:**
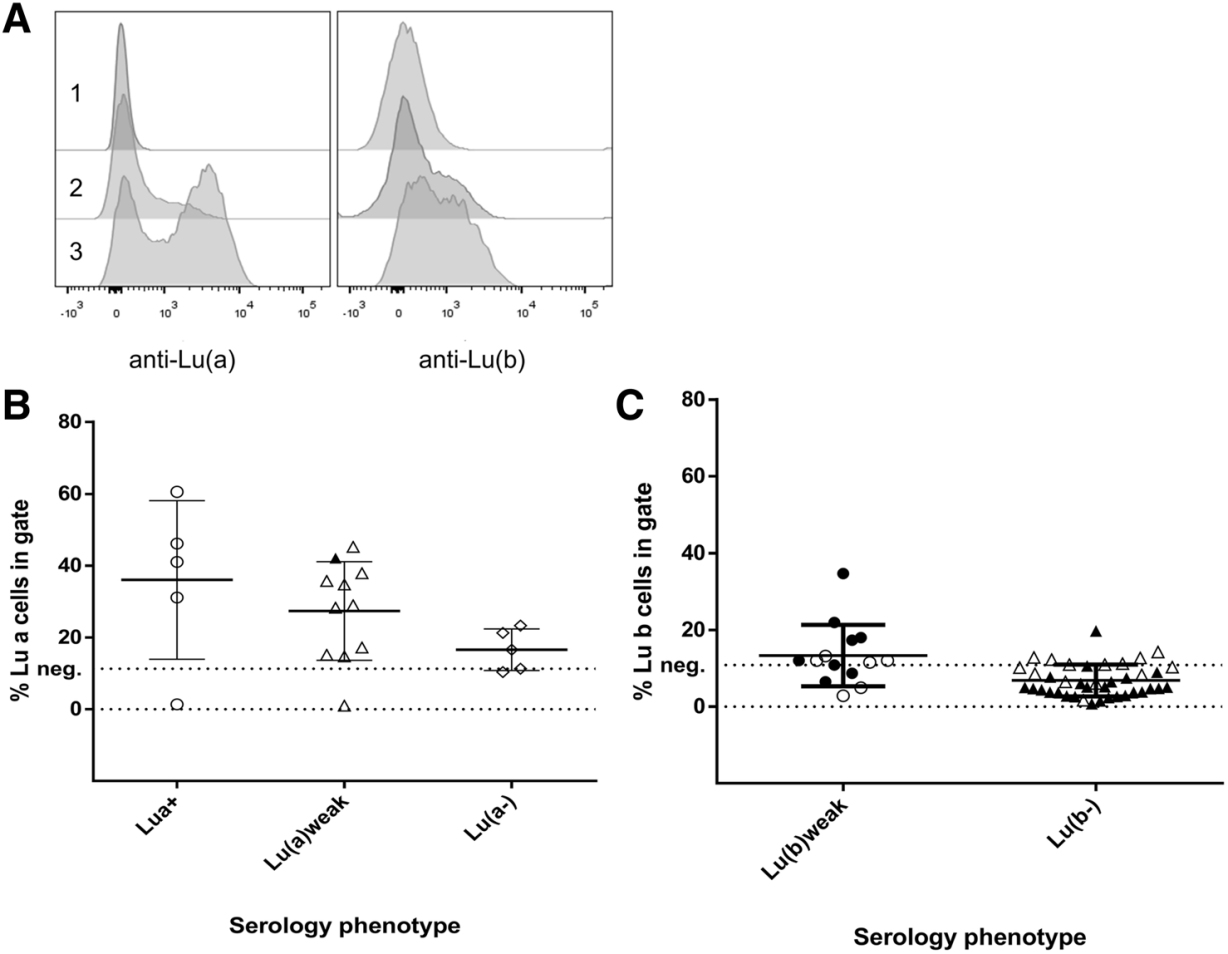
Typing erythrocytes for the Lu blood group by flow cytometry reveals a mixed field expression. **(A)** Representative Flow Cytometry LuA and LuB single stains experiments showing Lutheran expression as histograms for specific donors (1–3). Histograms show individuals negative for Lu(a, b) (1), weak expression of Lu(a, b) (2) and positive for Lu(a, b) (3). Note the negative Lutheran populations. **(B)** Graph shows a correlation plot between FCM (y-axis; % of positive cells) and serology typing (x-axis) for individuals carrying the Lutheran A gene. Dotted line represents the cut-off range set for Lutheran negativity Lu(a) < 11.30%. Serology was more sensitive in two cases, and FCM was more sensitive in three cases. Genotype was considered, open symbols resemble (heterozygous) LuAB carriers, closed symbols resemble (homozygous) LuAA carriers. Homozygous LuBB carriers were excluded. **(C)** Correlation between FCM and serology for individuals carrying the Lutheran B gene (with a cut-off for Lu(b) < 10.84%). Serology was more sensitive in 5 cases, and FCM was more sensitive in eight cases. Zygosity was considered, open symbols resemble (heterozygous) LuAB carriers, closed symbols resemble (homozygous) LuBB carriers (homozygous LuAA carriers were excluded).

A cohort of 55 Lu weak (n = 25) or negative (n = 30) donors were identified using standard agglutination techniques (Supplementary Table 1). Detection of the Lu blood groups by antibody-mediated agglutination results in different degrees of agglutination, even in Lu positive samples. This is termed a mixed field result, which can lead to difficulties in discriminating between Lu negativity or weak expression^18^. To facilitate negative/positive Lu calling a flow cytometry (FCM) assay was set up. Lu(a) and Lu(b) expression can be readily detected by FCM and presents as a dual peak of negative and positive erythrocytes (Fig. 1A), providing an explanation for the mixed field results in Lu agglutination assays. Of note, this negative non-agglutinating population is slightly smaller in percentage on young erythrocyte/ reticulocytes but does not change between the bulk of the erythrocytes and old erythrocytes (Supplementary Fig. 1, Supplementary Methods). The FCM based Lu detection enables to discriminate low/weak/negative Lu expression based on the percentage of Lu(a + b +) events and mean fluorescent intensity. Threshold values for negative/positive calling are indicated (Fig. 1B,C; Supplementary Fig. 2). Comparing serology data and FCM data, in 3 out of 55 cases serology typed positive while this was not in concordance with genotyping (MLPA) results for Lu(a) (Supplementary Table 1). In 11 out of 55 cases, serology typed negative for Lu(a) or Lu(b) while FCM could still detect traces of Lu(a) or Lu(b), which was in agreement with genotyping. In 7 out of 55 cases serology could pick up a weak Lu(a) or Lu(b) signal while FCM was negative with the threshold set (Fig. 1B,C). Lutheran antigens are notoriously very variable in strength partly due to the mixed field results and complicating serology. Indeed, adsorption and elution tests are sometimes required to detect weak Lua or Lub.

### Sequencing of Lu weak and negative donors revealed novel variants in *KLF1*

**Figure 2:**
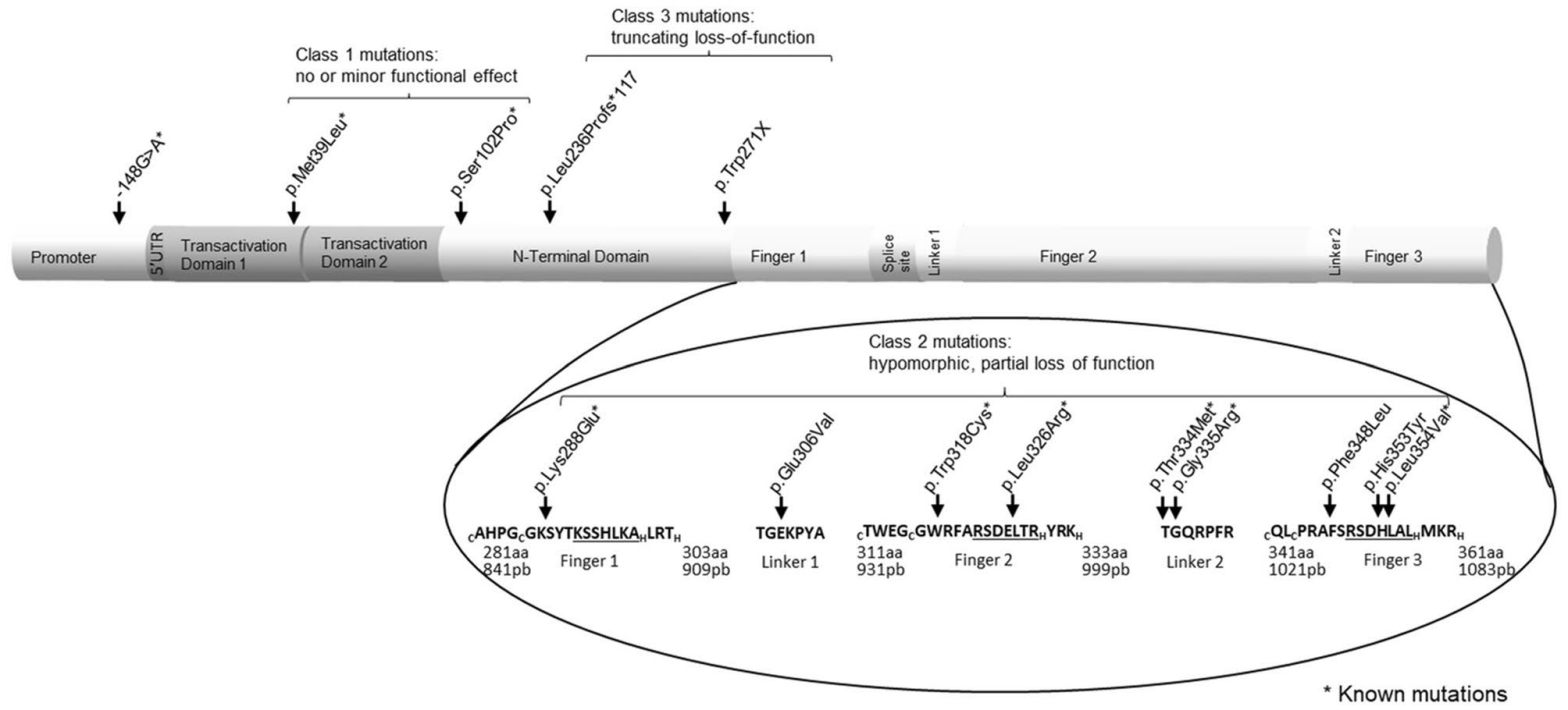
Sequencing KLF1 exons and promoter revealed known and novel variants. Summary of (novel) variants that were found in KLF1 as indicated in Supplementary Table 1. Schematic representation of the KLF1 gene, depicting the promoter, protein binding and the 3 Zinc-finger domains. Variants are indicated by arrows. Inlet zoom displays a detailed overview of the three Zinc-fingers. Letters in superscript indicate amino acids involved in Zinc atom binding. Underlined letters indicate amino acids directly involved in DNA binding. Previously described variants are marked by asterisk.

Besides the known variants in *BCAM* causing Lu(a−b−), variants in *KLF1* can also result in a Lu negative phenotype termed In(Lu)^2^. DNA was isolated from peripheral blood mononuclear cells and *KLF1* exons 1, 2, 3 and the promoter region 684 bp upstream of exon 1 were sequenced (Fig. 2 and Supplementary Table 2). Within the cohort of 55 Lu weak/negative donors assessed by percentage of positive erythrocytes using flow cytometry, 2/25 (8%) Lu weak donors and 24/30 (80%) Lu negative donors displayed heterozygous KLF1 exon of which 4 are compound heterozygous promoter variants (g.-148G > A, rs79334031)^19^. In total 14 different variants were found of which 6 were novel. These were assigned to the different KLF1 variant classes (1) (Supplementary Table 1). The compound heterozygotes KLF1 promoter variant (g.-148G > A, rs79334031) was found 4 times in Lu negative donors, additional variants in a KLF1 exon were found in these individuals. Of note, no variants were found in the KLF1 hypersensitive site 1 (EHS1) promoter region, located between g.-506 and g.-576, containing Cis-regulatory elements^20^. One borderline Lu negative donor (8.7% Lu(b) positive donor) contained the c.917A > T KLF1 variant. Novel class 3 variants included an insertion mutant c.704dupT (Leu236Profs*117) and a c.813G > A (p.Trp271X) in the protein binding domain. The c.704dupT causes a frame-shift resulting in Zinc-finger loss, which is replaced by nonsense sequence and a putative protein of similar size as KLF1. The p.Trp271X causes a premature stop in the N-terminal protein binding domain, resulting in a truncated form of KLF1. Novel point variants leading to amino acid substitutions belonging to class 2^1^ were exclusively found in the Zinc-finger domain (e.g. p.Leu326Arg, p.His353Tyr, p.Leu354Val). Both c.1003G > A and the novel c.1001C > T variants are located in the second linker, outside the second zinc finger (Fig. 2). Both variants alter residues that are highly conserved throughout evolution^21^. The c.1003G > A variant leads to a p.Gly335Arg substitution altering the glycine residue. Two 2nd linker variants at the p.Gly335 site have been described before: Thr334Lys and p.Thr334Arg (rs483352841) and correlated with HPFH^22^. The novel c.1001C > T variant leads to p.Thr334Met substitution and possibly causes an alternate start site in the linker 2 region of KLF1. Of note, one Sickle cell disease carrier was found in the Lu weak group (Supplementary Table 1, indicated by **).

### In(Lu) individuals display lowered CD44 expression compared to control and other Lutheran negative individuals

**Figure 3:**
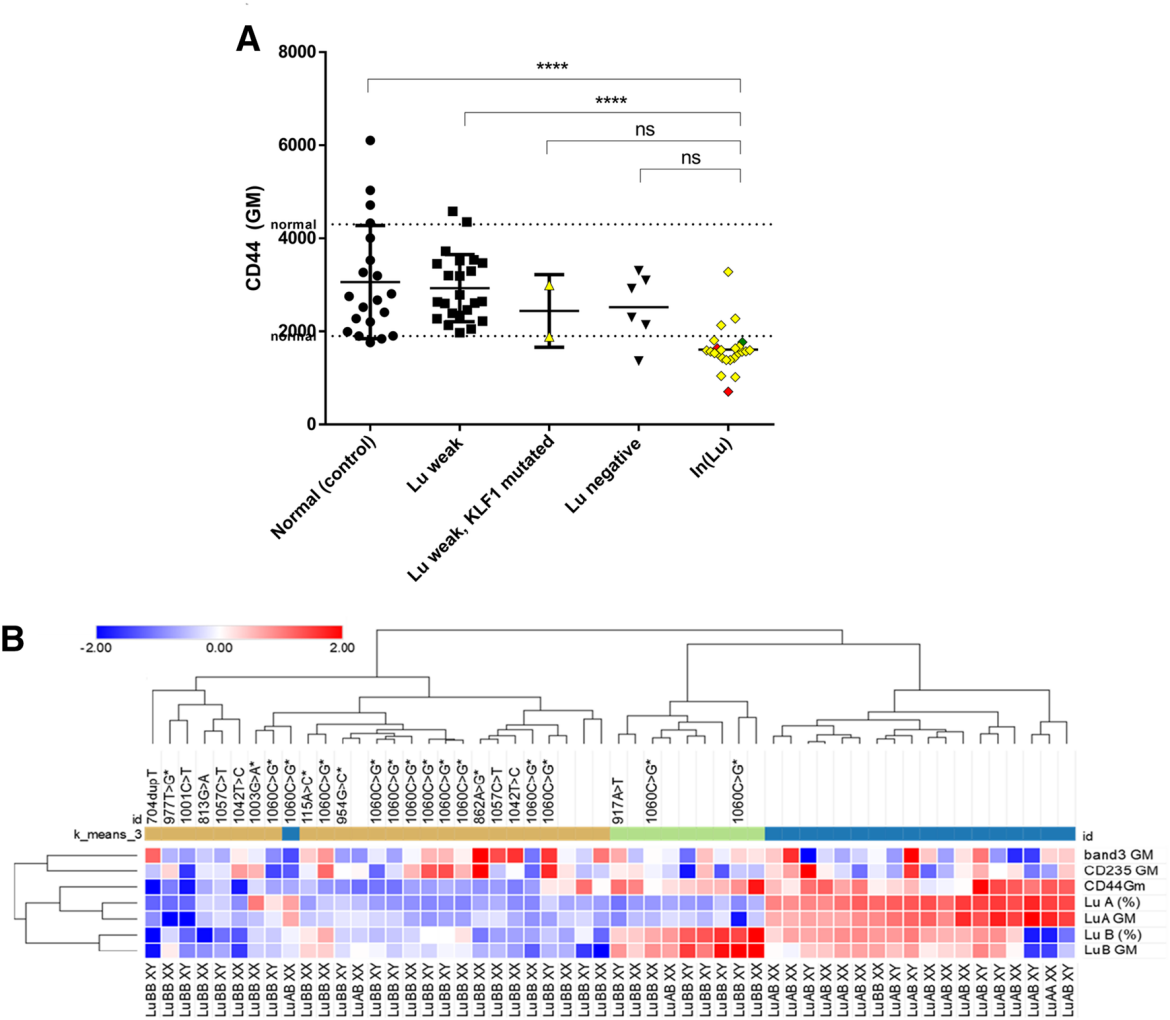
KLF1 mutants show significant decreased CD44 expression compared to control donors. **(A)** FCM MFI (mean fluorescence intensity) of CD44 on erythrocytes. Donors were grouped based on presence of KLF1 variants and Lu expression. The colors resemble the KLF1 variant class (on a scale of 1–3 indicating severity of the variant). Color nodes: green; class 1, yellow; class 2 and, red; class 3. The dotted line represents the normal value of CD44 expression. CD44 protein expression was plotted against the Lutheran phenotype as evaluated by FCM (Fig. 1). Dots represent the average MFI of three single individual measurements (*** indicates a P value of < 0.001, Tukey's multiple comparisons test). MFI is indicated as the Mean geometric Fluorescence Intensity of the signal. **(B)** FCM MFI data of CD44, CD235, Band3, LuB and LuA as well as the LuA + and LuB + percentage data was used to calculate individual z-scores after log2 transformation. The heatmap displays a hierarchical Euclidean complete clustering of columns and rows (CD44, LuB GM, LuB + , LuA GM and LuA + excluding CD235 GM and Band3 GM; *GM* geometric mean fluorescence) using Morpheus software (https://software.broadinstitute.org/morpheus). Lower part indicates the MLPA genotyping and upper part shows the various variants found within KLF1. K-means clustering was performed using 1000 interactions and N = 3 clusters. Of note, the figure shows 3 cases of LuAB MLPA typed individuals, without KLF1 variants, that display only LuA or LuB expression by FACS, which was verified by serology.

Variants in the transcription factor *KLF1* may lead to additional deregulation of *KLF1* target genes. CD44 (harboring the Indian blood group system) has been described as a KLF1 target gene^2^. In addition, variants in KLF1 have been shown to result in upregulation of fetal hemoglobin^9,23^ and correlate with increased adult hemoglobin A2^24^. We further characterized the Lu weak/negative cohort (including individuals without KLF1 variants), by measuring CD44, (harboring the Diego blood group system) and), GPA (harboring MNS antigens) and band 3 expression levels by FCM (Fig. 3A, Supplemental Fig. 3A,B). GPA and Band3 were used as controls to indicate that the changes in specific markers affected in In(Lu) are not caused by differences in the populations analyzed. Wild type, control erythrocytes display a variable scattered pattern of CD44 GM (Fig. 3A). Significantly lower expression of CD44 was found in donors displaying weak or negative Lu expression (Supplementary Table 5). In(Lu) donors showed the lowest average CD44 expression compared to control individuals. One of the 13 p.Leu354 variants, displayed a Lu(b) weak expression by FCM (but was Lu(b) negative by serology). In contrast, the same individual also showed a normal CD44 expression. Band3 and GPA membrane expression were not significantly different (Supplemental Fig. 3A,B, Supplementary Table 5). Hierarchical Euclidean complete clustering of FCM data using CD44, Lu(a) and Lu(b) z-scores segregated the Lu negative individuals with variants from the rest of the samples strengthening the link between KLF1 variants and low CD44 expression (Fig. 3B). The K-means clustering defines Lu(b) + cells is a separate cluster from Lu(a) or Lu(a,b), which may suggest correlations based on the Lutheran phenotype itself. Note that some heterozygous genotyped LuAB individuals are negative for either Lu A and/or Lu B antigens yet they do not harbor *KLF1* variants. It may be that Lutheran negativity is due to mutations in *BCAM*, which was not sequenced. As expression of band 3 and GPA segregated far away from the Lua or LuB, while CD44 clusters together with the LuA/B, this suggests their expression is correlated. Indeed, it has been reported by Singleton et al. that CD44 is a bona fide target gene of KLF1^2^.

### Mutations in KLF1 lead to changes in hemoglobin subunit distribution

**Figure 4:**
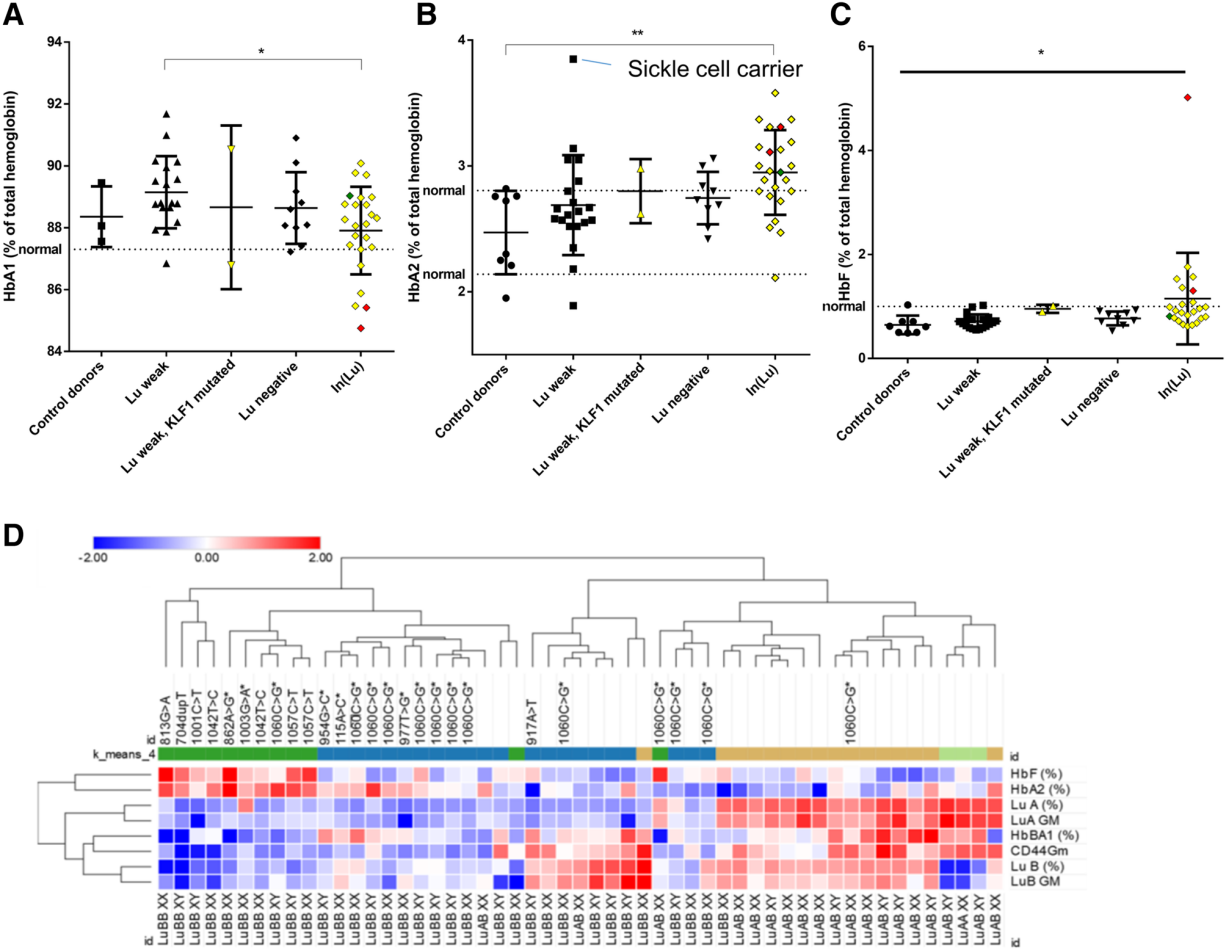
Donors that have KFL1 variants show an elevated Fetal Hemoglobin (HbF) and alpha 2 hemoglobin (HBA2) expression. Expression of HBA1 **(A)**, HBA2 **(B)**, HbF **(C)** was plotted against specific groups as indicated. The “normal” dotted base-line indicates the average of all control individuals. Donors were divided in groups based on their Lu expression and the presence of KFL1 variants. The graph shows the average hemoglobin expression per individual measured 3 times (* indicates P value of < 0.1; *** indicates a P value of < 0.001; Tukey’s multiple comparisons test was used for A and B, for C an unpaired student T-test on In(Lu) vs control donors groups was performed. **E**). The heatmap displays a hierarchical Euclidean complete clustering of columns and rows (*GM* geometric mean fluorescence) using Morpheus software. Lower part indicates the MLPA genotyping and upper part shows the various variants found within KLF1. K-means clustering was performed using 10,000 interactions and N = 4 clusters. Note that this clustering segregates the KLF1 variants that correlate with increased HbF from the rest of the samples (clustered to the left side of the panel).

Next we examined if the Lu weak and negative individuals display altered expression of specific hemoglobins by HPLC (Fig. 4, Supplementary Fig. 4A,B). Increased HbF and HbA2 expression in In(Lu) individuals was observed compared to normal controls and the remaining cohort (Fig. 4B,C, Supplementary Table 5). This was accompanied with a concomitant drop in HbA1 (α2β2) and resembles the HPFH phenotype (Fig. 4A). Some KLF1 mutants, such as the p.Trp271Ter and p.Lys288Glu show high HbF levels of 5% and 1.8% respectively. KLF1 variants that occur outside the DNA binding region also display phenotypic characteristics associated with In(Lu) and HPFH (Supplementary Table 1). Most of the In(Lu) individuals contained the class 2 c.1060C > G variant, which correlated with low CD44 expression, Lu negativity and elevated HbA2 levels but not with increased HbF levels. Euclidian hierarchical complete clustering of the HPLC and flow cytometry data of the Lutheran negative and Lutheran weak erythrocytes confirms the correlation between HbF and HbA2 as well as the correlation between individuals with KLF1 variants versus individuals with no KLF1 variants (Fig. 4D). Of note, within the Lu weak/negative donor population, the c.304 T > C polymorphism (rs2072597) shows a distribution of 45.5% for the homozygous TT allele, 47.3% for the heterozygous TC allele and 7.3% for the homozygous CC in concordance with frequencies observed in the European population (Supplementary Fig. 5). This polymorphism did not show a correlation with red blood cell membrane markers CD44, band 3, Lu(a), Lu(b), HbF or HbA2 expression levels. This is in agreement with the international society of blood transfusions workshop on red cell immunogenetics and blood group terminology which termed this particular KLF1 variant obsolete concerning In(Lu)^25^.

### Expression of KLF1 mutants in primary cultured human erythroblasts recapitulates the In(Lu) phenotype

**Figure 5:**
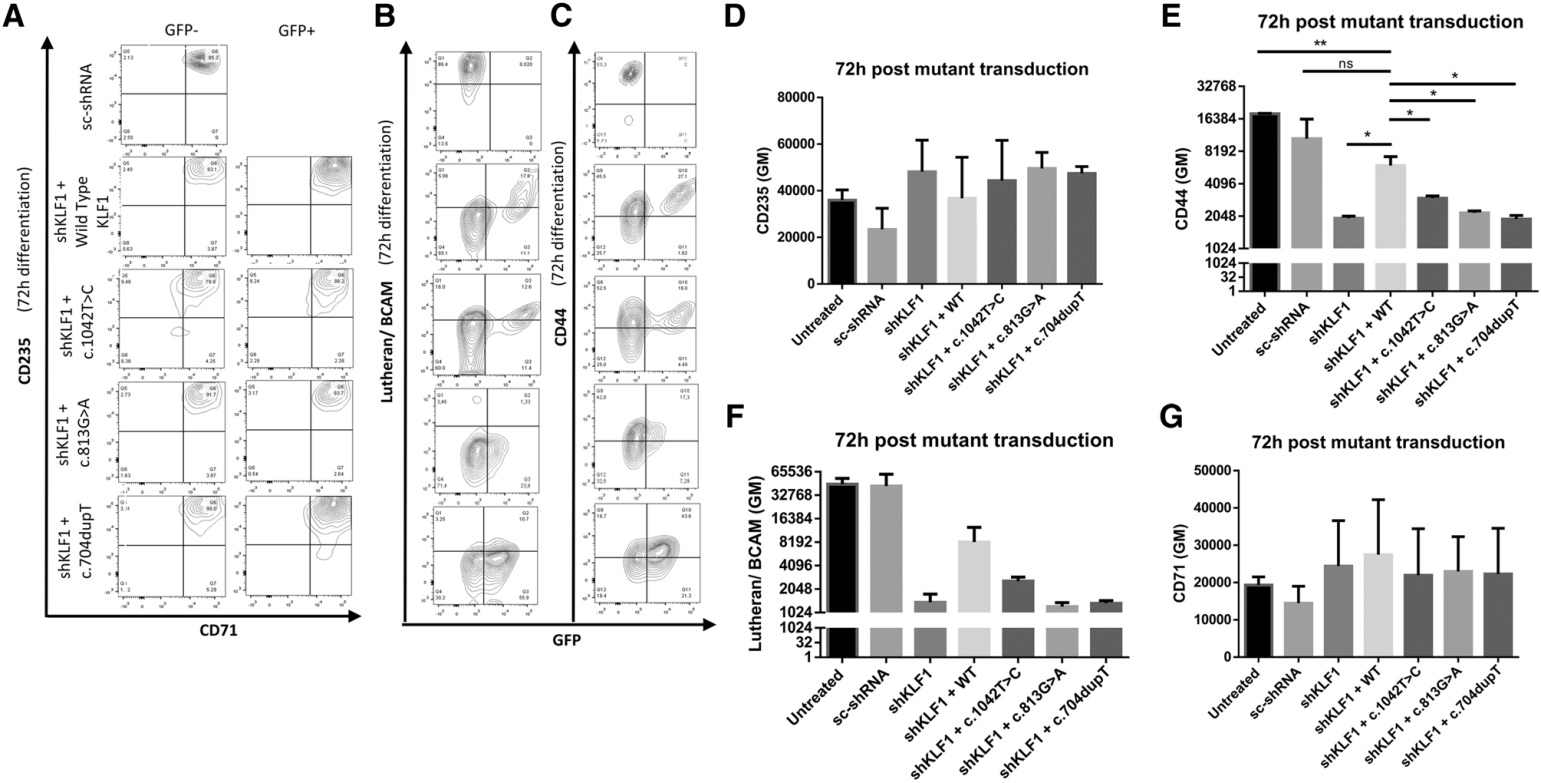
knockdown of endogenous KLF1 combined with re-expression of specific KLF1 variants show differential degrees of target gene expression rescue. Endogenous KLF1 was downregulated in primary human erythroblasts through transduction of short hairpin targeting 5’UTR of KLF1 mRNA. These cells were then selected by puromycin and transduced with wild type or mutant KLF1 and differentiated. **(A)** CD235 and CD71 expression levels do not overtly change upon knockdown or overexpression of wt or mutant KLF1. FCM plots show the differentiation state of erythroid cells at 72 h of differentiation. Cells in the GFP + plots (on the right axis of the plots) express mutant or wild type KLF1 and GFP- (left axis) do not ectopically express KLF1. Sh indicates co-expression with the shRNA against endogenous KLF1. **(B)** Lu/BCAM expression as depicted by FCM plots. Cells with lentiviral mediated expression of KLF1 wt or mutants are GFP + . **(C)** FCM plots shows the expression of CD44 in cells with or without an shRNA against endogenous KLF1 and with or without lentiviral induced expression of KLF1 wt or mutants (GFP +). **(D–G)** The expression (GM) of Blood Groups, CD235, CD44, Lu, andCD71 respectively, at 72 h of erythroid differentiation for each KLF1 mutant, wild type KLF1, in combination with shRNA against KLF1 or scrambled control.

**Figure 6:**
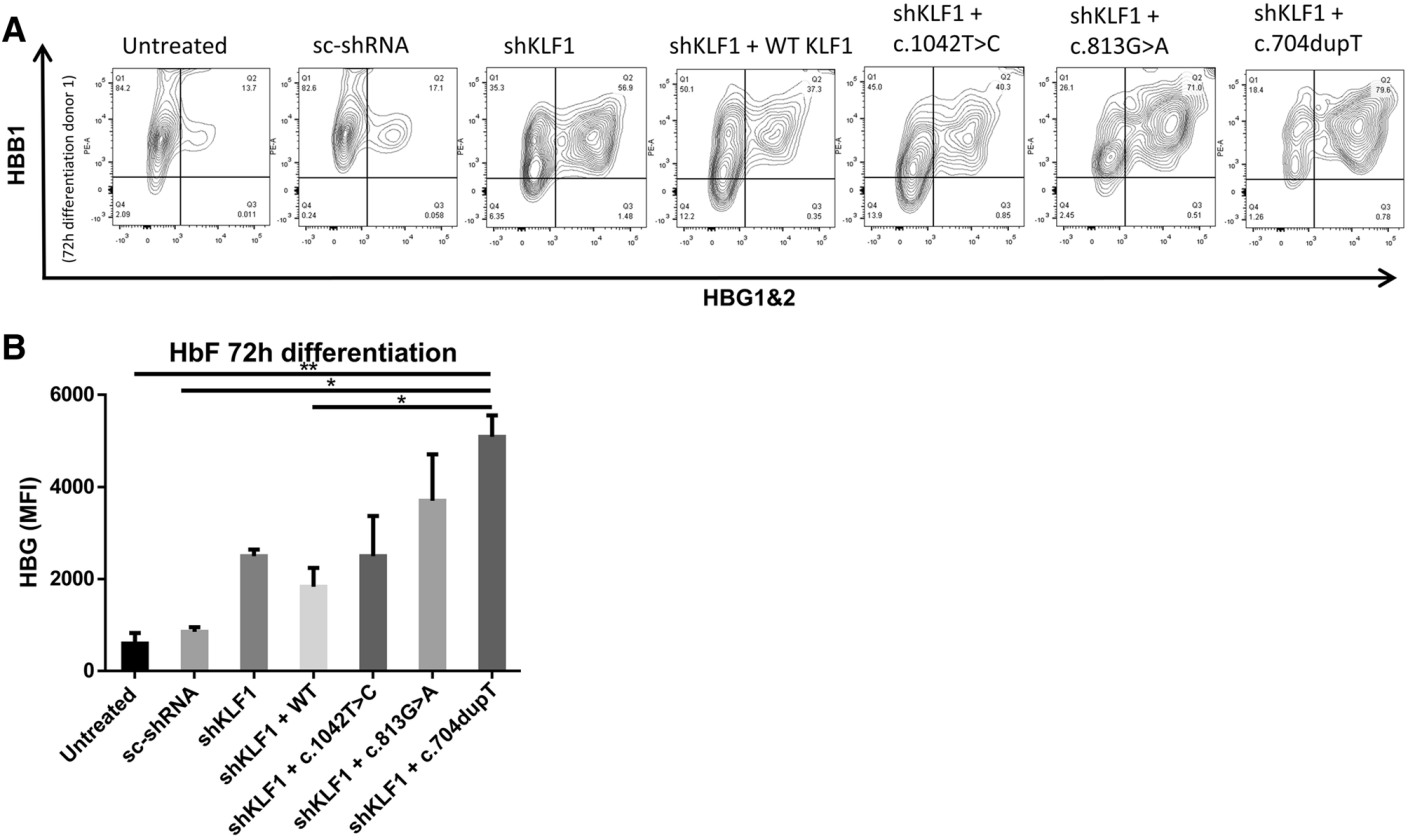
Effect of KLF1 variants on hemoglobin distribution during erythroid differentiation. **(A)** FCM plots show the HbA (anti-beta-globin; y-axis) and HbF (anti-gamma-globin; x-axis) distribution for one donor at 72 h of erythroid differentiation. Dot plots depicting shRNA against endogenous KLF1 (SH) together with KLF1wt or mutants were gated on only GFP + cells to exclude the contribution of non-ectopically expressed GFP- erythroid cells. **(B)** Mean fluorescence intensity of HbF expression at 72 h of differentiation. Stars indicate significance level (Tukey's multiple comparisons test).

The severity of the In(Lu) phenotype observed may be partly dictated by individual genetic differences. Indeed, this has been previously suggested to explain highly variable degrees of HPFH within a family harboring a single point variant in KLF1^10^. To control for these genetic influences and to study KLF1 variants within a similar genetic background, we ectopically expressed wild type KLF1 or KLF1 with novel variants whilst simultaneously knocking down (KD) endogenous KLF1 using 3’UTR recognizing shRNA’s in primary human erythroblasts cultured from three different Lu(a + b +) donors. For this transduction, we used the 2 class 3 variants (c.704dupT and the c.813G > A) that gave high HbF in our In(Lu) cohort, and the class 2 variant (c.1042 T > C) that gave a mild HbF expression. Endogenous KLF1 KD efficiency through puromycin selection of shRNA transduced primary erythroblasts was around 100% by Western Blot analysis (Supplementary Fig. 6). In addition, Western blotting showed expression of c.704dupT and c.813G > A, however, c.1042 T > C was barely detectable (Supplementary Fig. 6B). However, as the KLF1 mutants were expressed using a lentiviral construct containing KLF1-2A-GFP, GFP expression could be used to gate wtKLF1 or mutant expressing cells by FCM. The expression of Lutheran and other blood groups, hemoglobins and GFP was followed over time by FCM (Figs. 5 and 6). CD71 and CD235 expression is used to track differentiation state of erythroblasts^16,26,27^ and revealed a comparable differentiation progression between untreated, during the first 24–48 h (Fig. 5A,D,G and Supplementary Fig. 7A,D). KLF1 knockdown results in decreased expression of CD44 and BCAM, confirming transcriptional control by KLF1 (Fig. 5B,C,E,F, Supplementary Fig. 7B,C). Re-expression of KLF1 WT rescues CD44 and Lu expression but not to untreated or scrambled shRNA control sample levels. Expression of CD44 and Lu upon over-expression of KFL1 variants c.704dupT and c.813G > A comparable to knockdown of KLF1 and thus did not rescue. The c.1042 T > C variant led to an intermediate rescue of CD44 and Lu expression. Of note, the percentage of GFP positive cells did not change over the course of differentiation suggesting that KLF1 overexpression nor knockdown did not lead to a competitive disadvantage in the first 72 h of differentiation (Supplementary Fig. 7E). Upon further differentiation (120 h), the erythroid cells in which the endogenous KLF1 was knocked down showed a low percentage of viable cells and a further loss of CD44 expression compared to untreated or scrambled shRNA control cultures (Supplementary Fig. 8A–D), which confirmed the crucial role of KLF1 during erythropoiesis.

### Expression of mutant KLF1 in primary human erythroblasts recapitulates the HPFH phenotype

Next we evaluated if expression of KLF1 variants interfered with hemoglobin subunit expression and/or resulted in elevated HbF expression. HBG1/2 and HBB1 protein levels were measured on different time points of EBL differentiation using FCM (Fig. 6A). Expression of a scrambled shRNA did not lead to increased HBG1/2 expression in erythroblasts. However, erythroblasts transduced with shRNA against KLF1 show a significant increase of HBG1/2 expression during differentiation indicating the prominent role of KLF1 in repressing gamma globin expression (Fig. 6B). Re-expression of wild type KLF1 led to a reduction HBG1/2. Erythroblasts expressing the c.1042 T > C, c.813G > C and c.704dupT variants have higher averages of HBG1/2 when compared to EBL treated with scrambled RNA or wild KLF1. Only c.704dupT variant demonstrated significantly higher HBG1/2 levels. These data indicate that specific expression of KLF1 mutants in primary erythroblasts can cause HPFH.

## Discussion

Here we described novel variants in the *KLF1* gene that result in HPHF and in all cases causes dominant In(Lu). Interestingly, FCM revealed that Lu positive donors always display a negative population, which explains the mixed field observed in Lu(a + b +) donors upon agglutination based serology. The ratio is different between individuals but maintained upon erythrocyte aging. Stringent gate settings aid separating Lutheran negative individuals from extremely low expressing individuals thereby facilitating selection of better matched blood upon transfusion when Lutheran negative blood is necessary. Of note, since In(Lu) individuals are characterized by carrying not only reduced expression of antigens in the Lutheran system but also significantly reduced P1, In^b^, and AnWj antigens, alloimmunization by these antigens may also not be expected^2^. However, the thresholds set by FCM here, may still not discriminate extremely low Lutheran expressing RBC from total negative erythrocytes. Thus, although In(Lu) blood donors may score Lu(a-b-) on serology and FCM tests, they may still contain traces of Lu(a +)/Lu(b +). Importantly however, the thresholds used here segregated the Lu negative individuals with KLF1 variants from low Lu expressing individuals without KLF1 variants. It should be noted however, that transfusion dependent recessive Lu(a− b−) patients might have a risk of developing alloimmunization when receiving blood from In(Lu) donors, however the chance of this actually occurring is extremely low.

In 24 out of 30 Lu negative individuals (80%) variants in *KLF1* were found, these individuals can thus be classified as In(Lu), and, the frequency found here is comparable to that of other studies^28^. To explain the Lu negative phenotype in the remaining 6 out of 30 individuals, more research needs to be done. A possible explanation may come from homozygous autosomal-recessive mutations in the *BCAM* gene, or hemizygosity for a recessive X-linked *GATA1* mutations that have previously been shown cause Lu negative phenotypes^6^. From all individuals harboring KLF1 variants, 9 (c.704dupT, c.813G > A, c.862A > G, c.1001C > T, c.1057C > T, c.1003G > A, and in two out of 13 c.1060C > G cases) show elevated levels of HbF. Of these, all class 3 variants and both individuals carrying the c.1057C > T variant in different donors, showed elevated HbF, indicating that this is a mutant specific effect. 20 individuals display elevated HbA2 (> 2.8%). Indeed, class 2 and 3 KLF1 variants have been shown to correlate with elevated HbA2 expression^24,]^^29^. According to the recently proposed KLF1 mutant classes^1^, we have identified 4 novel class 2 and 2 novel class 3 variants. Over-expression of a selection of mutants in a KLF1 knockdown setting recapitulated the observed phenotypes in KLF1 mutant donors, providing a model to study specific KLF1 mutants in the same genetic background and underscoring the role that KLF1 plays in regulation of erythroid surface proteins and specific globin expression.

KLF1 functions in a large range of processes during erythropoiesis and specific variants within KLF1 result in a range of different phenotypes and diseases^1^. This underscores the pleiotropic effect of KLF1 in wt and mutant settings. In addition, KLF1 is also involved in regulating globin expression either directly through transcriptional activation of the beta globin gene or indirectly through repression of gamma globins, e.g. through BCL11A or LRF^10,11,30^. Haploinsufficiency or expression of KLF1 variants may induce persistence of fetal hemoglobin. Interestingly, families with the same KLF1 mutant display significant differences HbF levels (range between 2 and 20%), indicating additional contributing factors independent or dependent on KLF1^31^. Here we also observed elevated HbF levels in some but not for all In(Lu) class 2 variants underscoring either the pleiotropic effect of these different mutants or the confounding consequence of other genetic variation between the In(Lu) individuals^1^. Due to genetic variation it is relatively difficult to segregate the mild effect of other specific KLF1 variants on HbF regulation from other confounders. As shown here, the re-expression of KLF1 variants in the same genetic background is useful to compare and understand the contribution of different KLF1 mutants to target gene regulation without completely understanding underlying confounding genetic parameters. For instance, expression of the KLF1 c.1060C > G Zn-finger variant (p.Leu354Val, rs1397962733; found 13 times) presented with elevated HbF in only 2 out of 13 donors and decreased CD44 12 out of 13 donors. Despite its high frequency, it has not been reported before putatively reflecting a founder effect within this Dutch cohort. This missense variant was never bi-allelic and homozygosity may be incompatible with life.

One out of the two class 3 variants (p.Leu236Profs*117) expressed the lowest CD44 levels found in this cohort. This finding indicates that an extremely low CD44 expression may be a variant specific effect for class 3 variants. The class 3 frameshift variant p.Leu236Profs*117, and the premature stop variant p.Trp271X, both result in the loss of the three Zinc-finger domains. The p.Trp271X variant displays the highest increase in HbF. They phenotypically match previously reported class 3 variants, p.Ser270X, p.Pro190LeufsX47 and p.Arg319GlufsX34^2,32^, which described nonsense mediated decay of KLF1 RNA resulting in haploinsufficiency. The effects of missense variants are much harder to predict, for example the heterozygous class 1 p.Ser102Pro (c.304 T > C) variant was found in 26 of 55 times in our cohort and does not seem to have a significant effect on CD44 expression as suggested previously^25,33^ (Supplementary Fig. 5, Supplementary Table 5). In contrast, the compound heterozygous promoter variant (c.-148G > A), p.Met39Leu (c.115A > C), p.Lys288Glu (c.862A > G), p.Trp318Cys (c.954G > C), p.Leu326Arg (c.977 T > G), p.Phe348Leu (c.1042 T > C), p.His353Tyr (c.1057C > T), all show low CD44. The c.954G > C and c.977 T > G variants had lowered CD44 levels, which is in agreement with a previous study^34^. Similarly the c.977 T > G variant showed increased HbA2 but no elevated HbF^34^. As expected no changes in expression of surface proteins Band3 and GPA in the In(Lu) cohort were observed.

Even though the class 1 p.Met39Leu (c.115A > C) variant presented with lowered Lu and CD44 expression in this study, it was previously reported as possibly neutral as mouse Klf1 contains a leucine at this position^10^. The compound heterozygous KLF1 promoter variant (c.-148G > A, rs79334031) has been described before to be associated with increased HbF levels^19^. It has previously been shown that CD44, *HBB* and *BCAM* are down regulated after in vitro culture of In(Lu) erythroblast^2^, which was a result of decreased binding to the promoters of these genes^12^, however, this was never shown on protein level. By in vitro knock down of endogenous wild type KLF1 and re-expression of mutated KLF1 co-expressed in healthy human erythroblasts we studied the effect of the novel frameshift variant p.(Leu236Profs*117) (c.704dupT), nonsense variant p.Trp271X (c.813G > A), and missense variant p.Phe348Leu (c.1042 T > C). Our in vitro experiments confirmed the in vivo found phenotypes. In contrast to haploinsufficiency, knockdown of KLF1 always resulted in similar phenotypes irrespective of the donor, indicating that genetic variation is of minor influence upon significant KLF1 knockdown.

In conclusion, findings described here suggest that all found variants lead to BCAM negativity and decreased CD44. Class 3 variants showed an intensified decrease of CD44. Two out of the eight type 2 variants and all class 3 variants resulted in HPFH, indicating the variant specific effects. The c.1060C > G variant showed variability between individuals concerning hemoglobin expression and blood group distribution, indicating that undetermined donor differences play a role. Our findings were confirmed by ectopic expression of specific KLF1 mutants that did not rescue BCAM and CD44 expression upon knocking down endogenous KLF1, indicating that these mutants were responsible for the In(Lu) phenotype. We found that, ectopic mutant expression gave a higher HbF level compared *KLF1* knock down conditions. This effect can be further explained through very high HbF levels (> 70%) found in a child where both parents were carriers of class 3 KLF1 variants^35^. Furthermore, in previously reported mouse studies, where the combination of KLF1 haploinsufficiency and a complete absence of BCL11A resulted in γ-globins are not expressed to the full extent^36^.

Ectopic expression of KLF1 mutants in 3 different donors showed no confounding effects. The fact that only 1 patient has been described to date that is null for KLF1 displaying severe anemia and complete transfusion dependency indicates the importance of KLF1 in erythropoiesis^35^. It is intriguing to observe such differential effects of KLF1 haplo-insufficiency on a selection of target genes like *CD44* and *BCAM*, while clearly not detrimental to the donor or affecting expression of other known KLF1 target genes^37^. Whether this can all be explained by lowered binding affinity of KLF1 for promoter regions or that competition at the level of cofactor recruitment plays a role remains to be investigated. Using over-expression of specific mutants in human pro-erythroblasts as shown here may help to uncover the underlying mechanisms.

In conclusion, we have found 6 novel KLF1 variants that lead to In(lu) phenotype. These mutants have subtle but clear effects on the expression of globin subunits and CD44 in erythrocytes, which were confirmed during in vitro erythropoiesis for KLF1p.(Leu236Profs*117) (c.704dupT), p.Trp271X (c.813G > A), and p.Phe348Leu (c.1042 T > C). Finding novel KLF1 mutations that lead to in(lu) is important in blood group profiling of individuals.

## Supplementary Information

Supplementary Information.
