# Supplementary material for: Novel variants in Krueppel like factor 1 that cause persistence of fetal hemoglobin in In(Lu) individuals
Source: Sci Rep. 2021 Sep 17;11:18557. doi: 10.1038/s41598-021-97149-y (PMC8448862; doi:10.1038/s41598-021-97149-y)
Supplement: Supplementary file 1 — Supplementary Information. [file 41598_2021_97149_MOESM1_ESM.pdf]

Supplementary information

Novel variants in Krueppel Like Factor 1 that cause Persistence of Fetal Hemoglobin in In(Lu) individuals

Jesse Eernstman[1], Barbera Veldhuisen[2], Peter Ligthart[2], Marieke von Lindern[1], C. Ellen van der Schoot[2], Emile van den Akker[1*]

Supplementary Methods

Percoll density gradient centrifugation of RBC

Three RBC fractions were generated by Percoll density gradient centrifugation from a Lu(a+b+) donor. Erythrocytes were diluted 1:1 with PBS and loaded on 15mL Percoll and spun down (zero brake).Pellet was collected and washed with PBS. Density gradient centrifugation was done by adding the high density RBC pellet to 40mL Urografin (30%), 25mL distilled water and 35mL Percoll. The suspension was then centrifuged at 20000 rpm for 20 minutes. Subpopulations were clearly separated and collected, stained analyzed by flow cytometry.

Supplementary Figures

Supplementary Figure 1. Lutheran expression during erythrocyte aging is relatively stable. Three RBC fractions were generated by Percoll density gradient centrifugation from a Lu(a weak, b+) donor as previously reported [D'Alessandro, 2013 #1906]. The top of the gradient consists of the youngest/immature RBC and age of RBC increases with increasing density. Subpopulations were collected as indicated in **(A)** with H=young RBC, M= medium aged RBC, L=old RBC. Subpopulations were stained for Lu(b) and Lu(a) and analyzed by flow cytometry. The dot plot displays the strategy to gate erythrocytes and the histogram offset for the Lutheran a and b staining is shown in **(B)**. **(C)** A small decrease in the MFI of Lu(a) and Lu(b) occurs during RBC aging but is similar between donors. Percentage of cells expressing Lu(a), Lu(b) for each gradient, medium and low fractions show a decrease of ~10% Lu(b) cells **(D, E)**. Lu(a), Lu(b) MFI for medium and low fractions show a decreased MFI of Lu(a) and Lu(b) **(F, G)**. The data is suggesting that Lutheran expression decreases slightly during RBC aging but is relatively stable.(*P value <0.1; ** P value of <0.01; *** P value of <0.001; Tukey's multiple comparisons test).

Supplementary Figure 2. Lu(a) and Lu(b) expression measured by Flow cytometry. (A) Serology Lu negative, low and weak donors were analyzed by Flow cytometry. Values represent percentages of Lu(b) (y axis) / Lu(a) (x axis). The box indicates the threshold of Lu detection limit. Threshold values were determined by analyzing donor red blood cells by Flow cytometry on cells that were positively serology typed. Whether an individual was rendered Lutheran negative, was based on properties such as presence of KLF1 mutations, and its MLPA score and Lutheran expression by FCM (S Tables). **B**) Indicates the correlation between the percentage and MFI for LuA (upper panel) and LuB (lower panel). The regression coefficient is given (R^2). **(C)** Geometric mean fluorescence intensity values of Lu(a) vs Lu(b) accompanied by the correlation between the percentage and MFI for Lu(b) **(D)**.

Supplementary Figure 3. (A) FCM MFI (mean fluorescence intensity) of BAND3 on erythrocytes. Donors were grouped based on presence of KLF1 variants and Lu expression. The "normal (control)" group resemble healthy blood donors. The "Lu weak" group resemble blood donors with weak Lu(a) or Lu(b) antigen expression. The "Lu negative" group resemble blood donors with no Lu(a) and Lu(b)

antigen expression and were no KLF1 variants were found. The colors resemble the KLF1 variant class (on a scale of 1-3 indicating severity of the variant). Color nodes: green; class 1, yellow; class 2 and, red; class 3. **(B)** Glycophorin A (GPA, CD235) protein expression was plotted against the Lutheran protein phenotype as evaluated by FCM (Figure 1).

Supplementary Figure 4. A, B) Hemoglobins were measured by HPLC and the percentage of specific hemoglobins was calculated by integrating the peak surface area. Examples of a donor with normal **(A)** and elevated **(B)** HbF expression are shown. The HbF, HBA1 and HBA2 peaks are indicated (arrows).

Supplementary Figure 5. The rs2072598 Ser102Pro KLF1 single nucleotide polymorphism does not influence CD44 expression. CD44 expression (percentage as measured by flow cytometry) within homozygous and heterozygous individuals for SNP rs2-72597 is shown for all *In(Lu)* and Lu weak donors combined. This 304T>C results in a Ser120Pro mutation.

Supplementary Figure 6. Knockdown of KLF1 in cultured human erythroblasts. (A) EBL were transduced with a shRNA against the 5'UTR of KLF1 (lane 1) or sequentially transduced with first the shRNA and then with KLF1wt (lane 2) as described in material and methods. **(B)** EBL were lentivirally transduced with KLF1wt and specific mutants as indicated (the lentiviral construct contains the KLF1 ORF coupled to a 2A sequence followed by GFP). Note that the expression levels of KLF1 *c.813c>g* lead to a truncated protein of 35kDa. Anti-RhoGDI is used to check equal loading.

Supplementary Figure 7. Antigen expression during the course of erythroblast differentiation (A-D) The expression (geometric mean (GM)) of Blood Groups, CD235, CD44, Lu/BCAM and, CD71 respectively, over the course of erythroid differentiation for each KLF1 mutant, wild type KLF1, in combination with shRNA against KLF1 or scrambled control as indicated in the legend caption. **(E)** Percentage of GFP positive cells (respective to total cell population), over the course of differentiation.

Supplementary Figure 8. KLF1 knockdown results in severely decreased numbers of viable cells 120 hours after differentiation initiation. A) Representative dot plot displaying side scatter and

forward scatter dot plot of cultures with knock down of endogenous KLF1 using short hairpin RNA against the 5'untranslated region of the KLF1 mRNA. Note the severely diminished amount of viable cells compared to untransduced **(B)** or scrambled shRNA **(C)** controls. Within the viable cells, KLF1 KD cells show lower CD44 expression levels compared to untransduced cells and scrambled shRNA **(D)**.

Supplementary Tables

Supplementary Table 1. Overview of *In(Lu)* individuals depicting identified KLF1 mutations, expression levels of erythroid membrane proteins (FCM) and globin subunits (HPLC). Erythrocyte Lu(a) and Lu(b)expression levels by FCM compared to serology scoring. The colors resemble the KLF1 mutation class.

Supplementary Table 2. KLF1 primers used for sequencing the *KLF1* gene

Supplementary Table 3. Statistics accompanying Figures

Supplementary Table 4. Antibodies

Supplementary Table 5. Mutagenesis primers to generate KLF1 mutants

Supplemental Figure 1

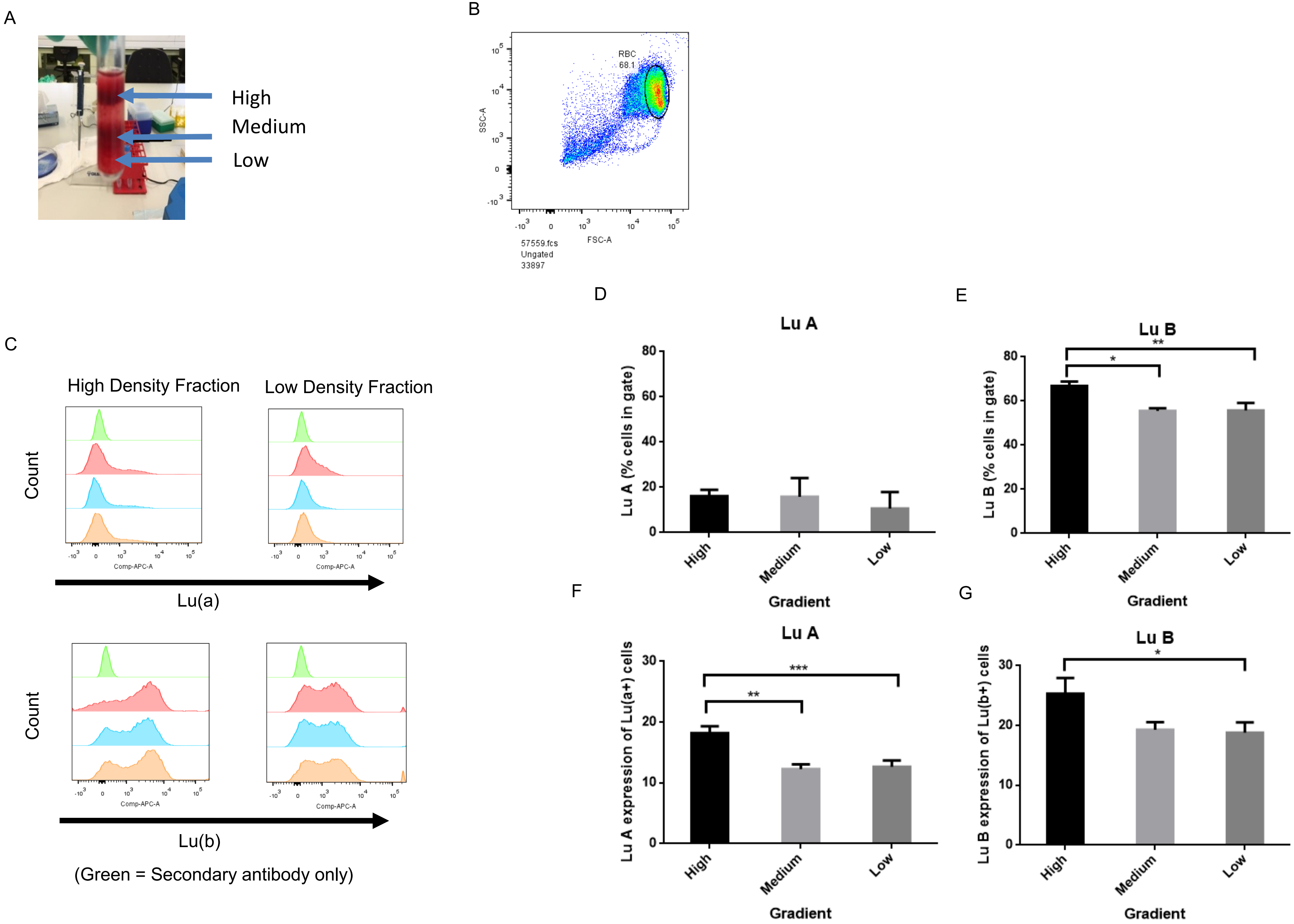

Supplemental Figure 2

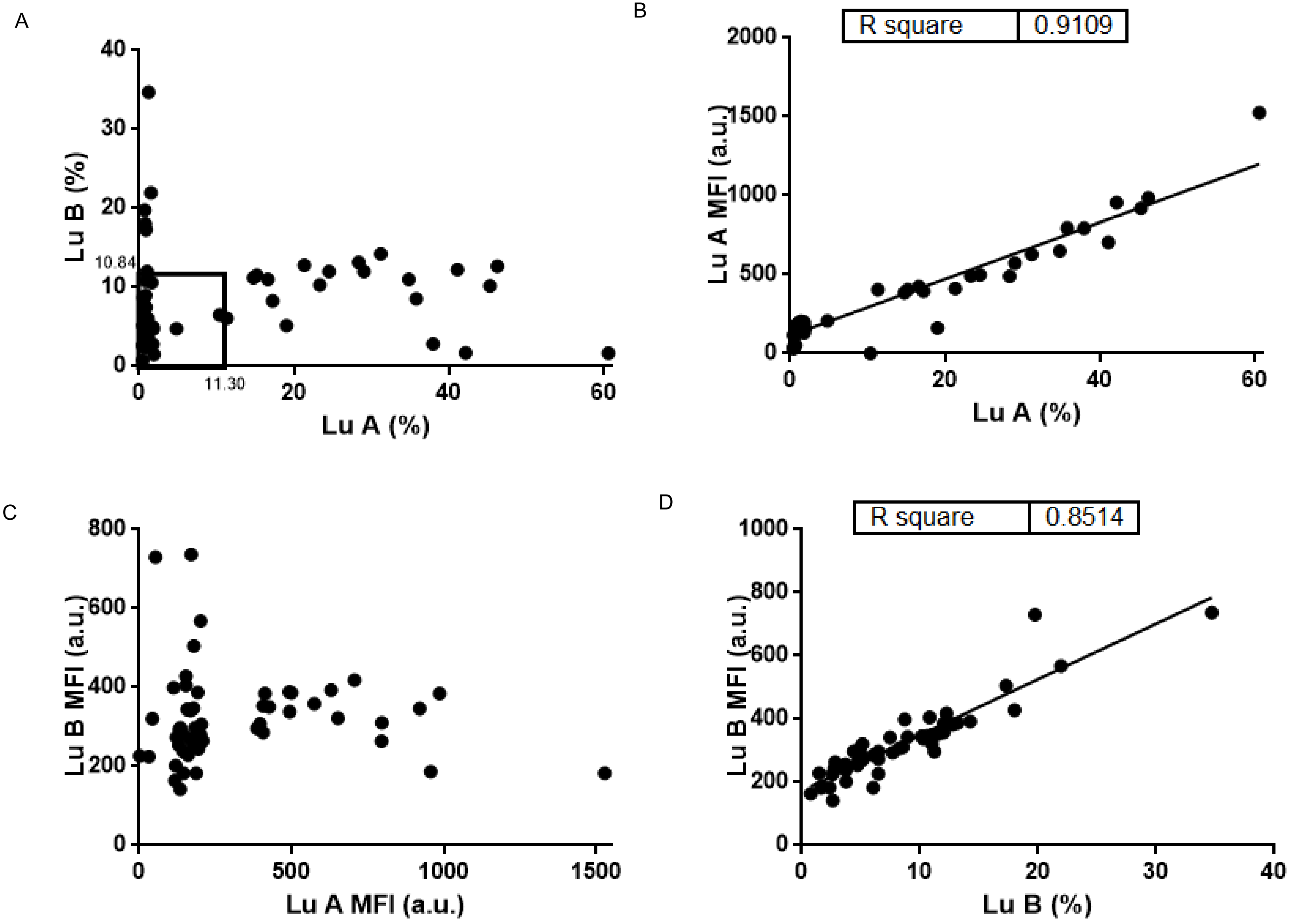

Supplemental Figure 3

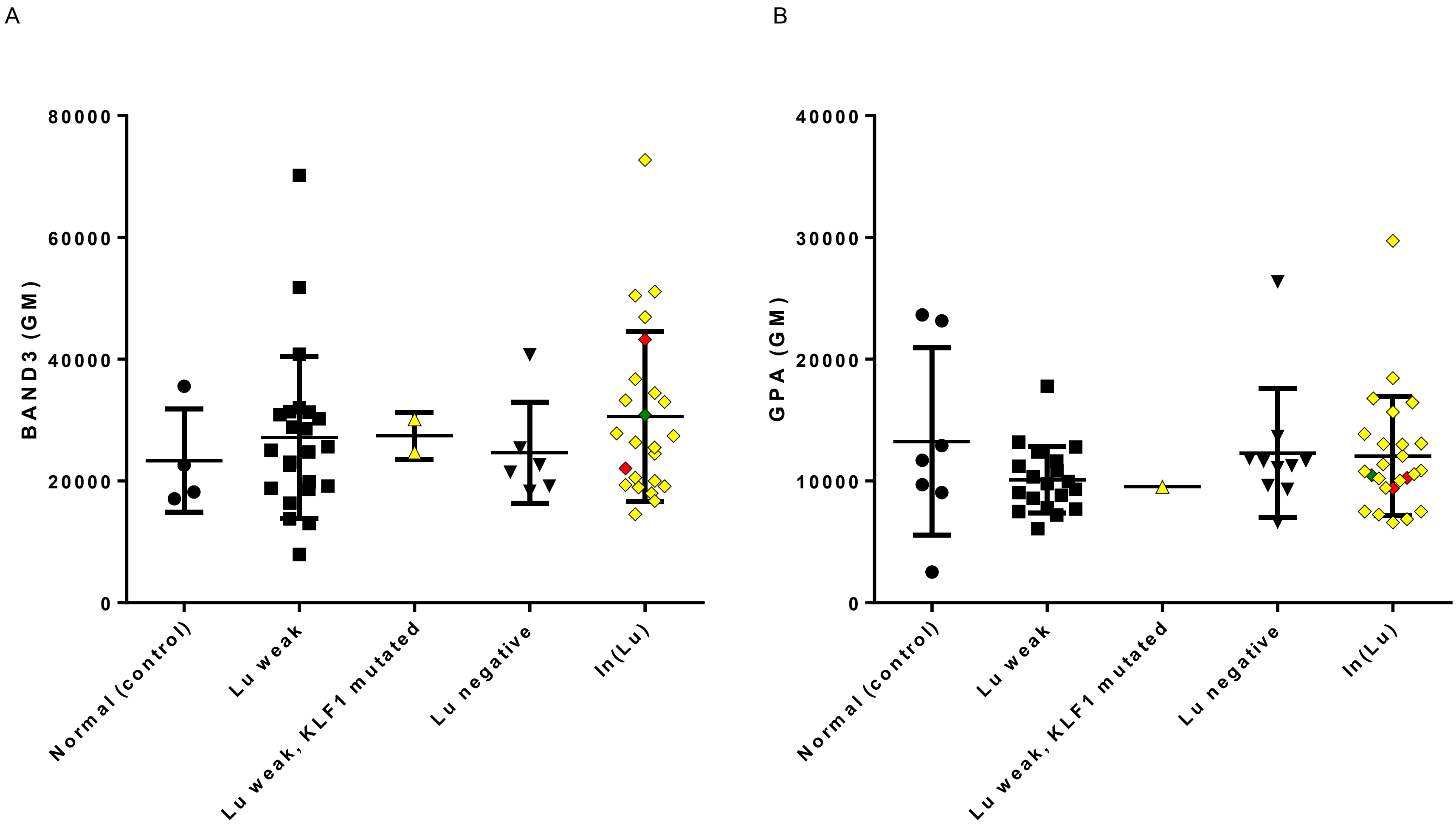

Supplemental Figure 4

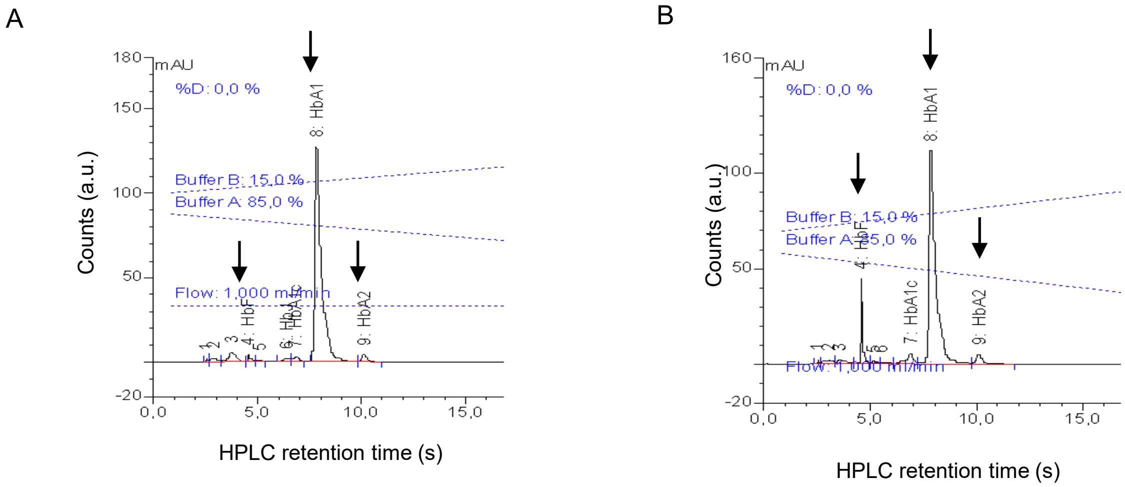

Supplemental Figure 5

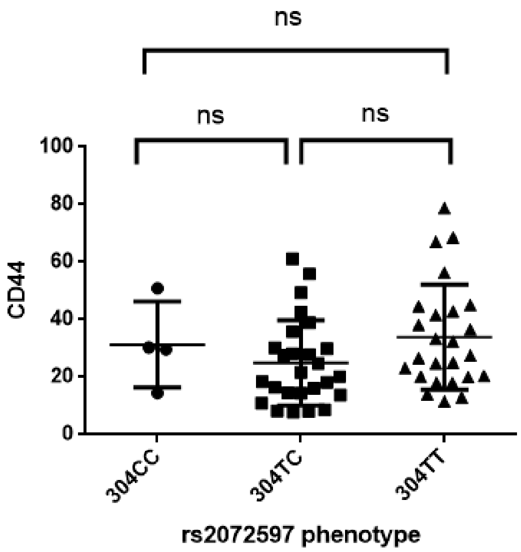

Supplemental Figure 6

A

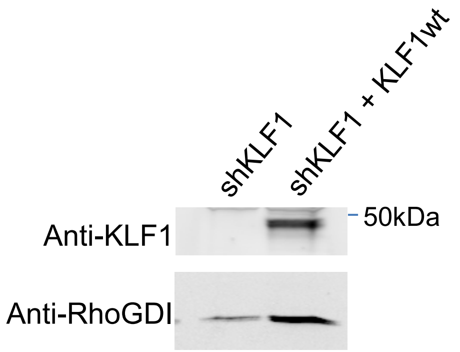

B

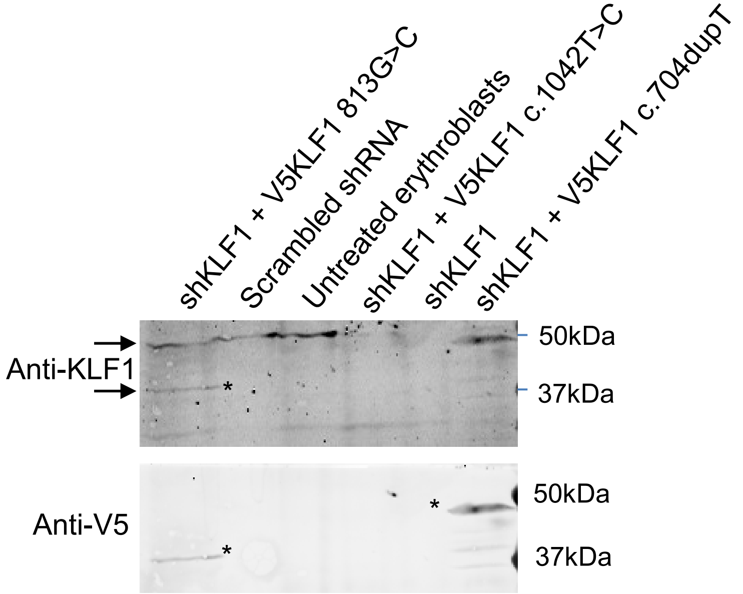

Supplemental Figure 7

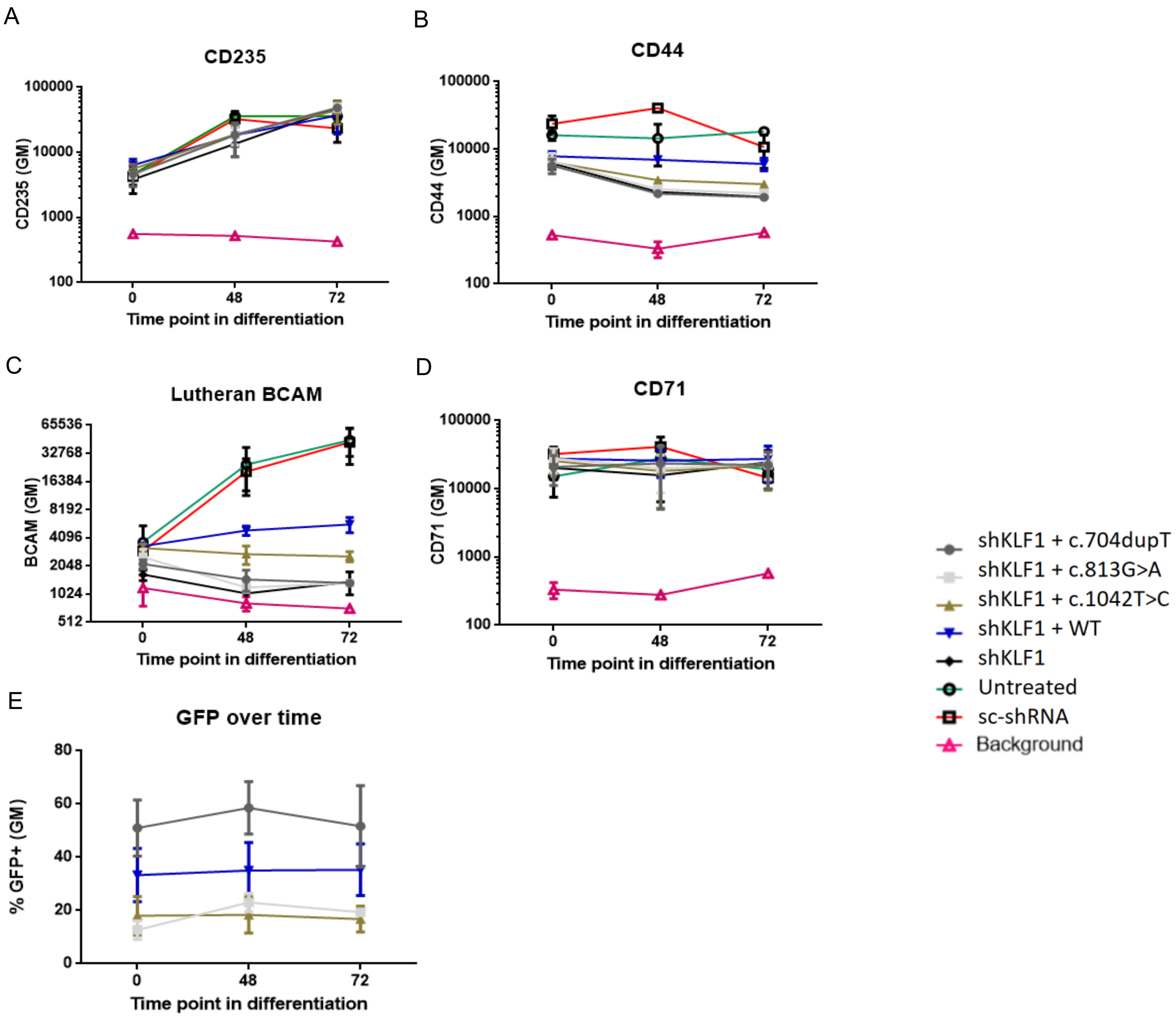

Supplemental Figure 8

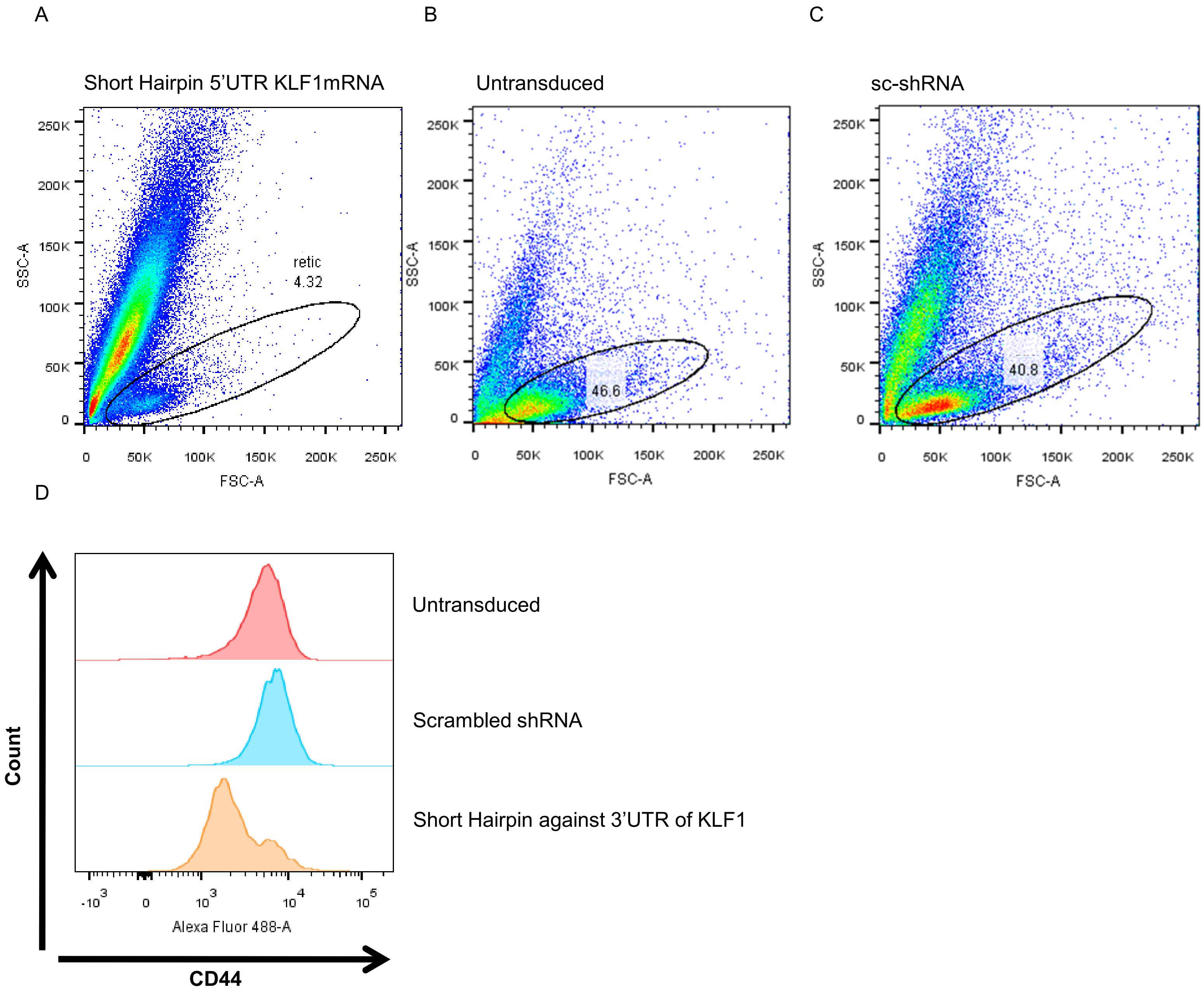

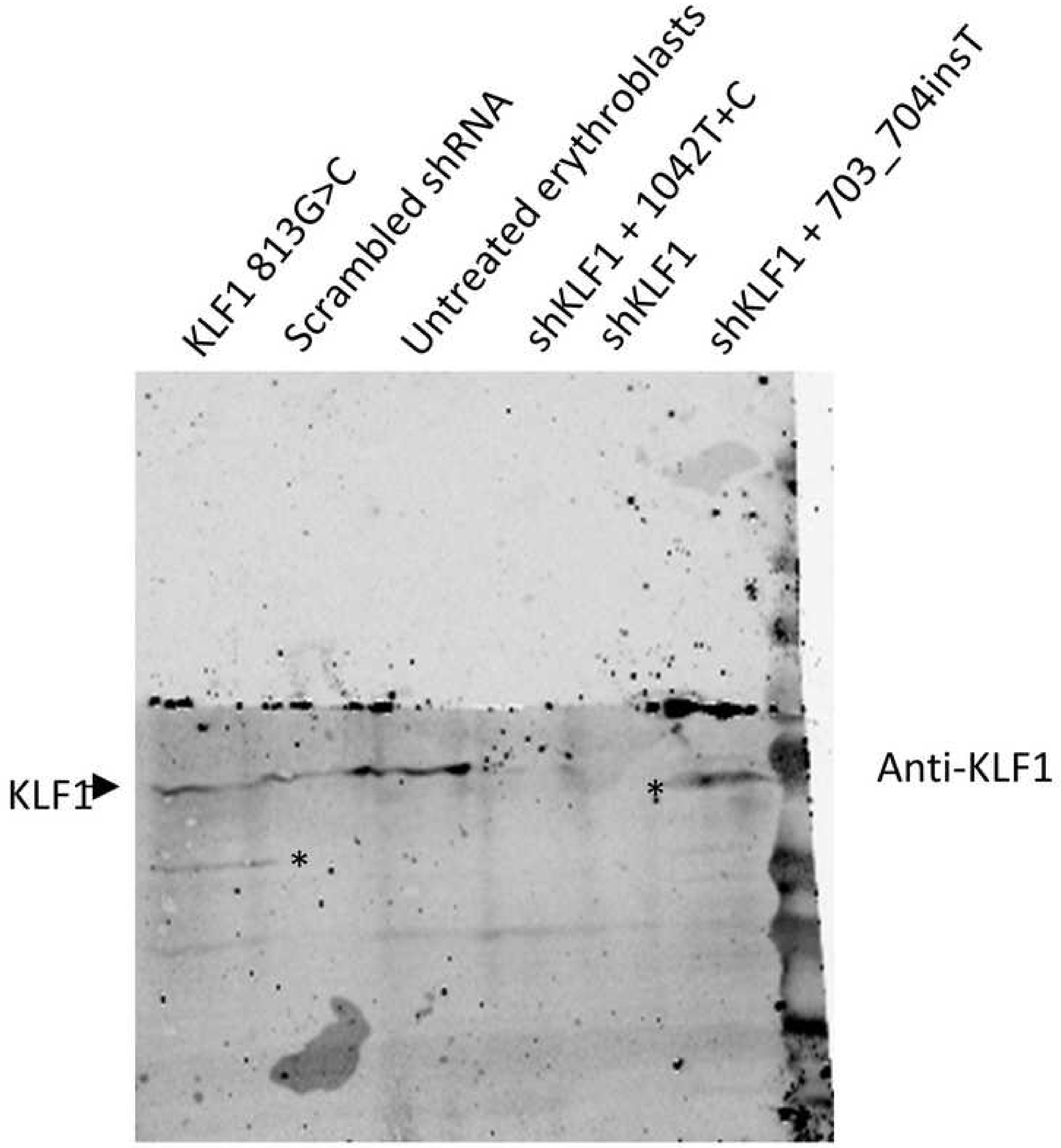

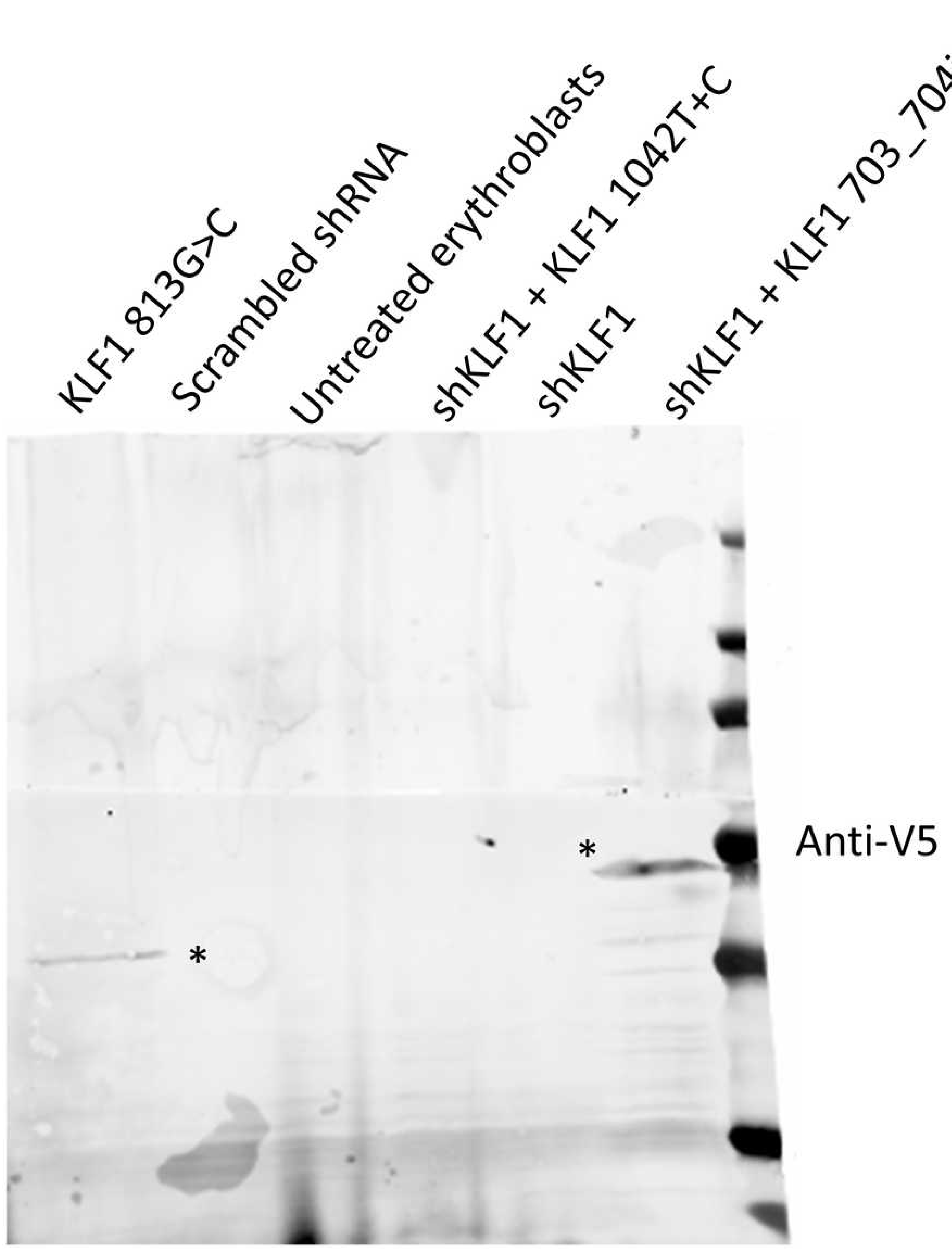

of note, the top of these membranes were cut off to stain hence the abrupt change. bottom part was stained with anti-KLF1 or anti-V5 as indciated

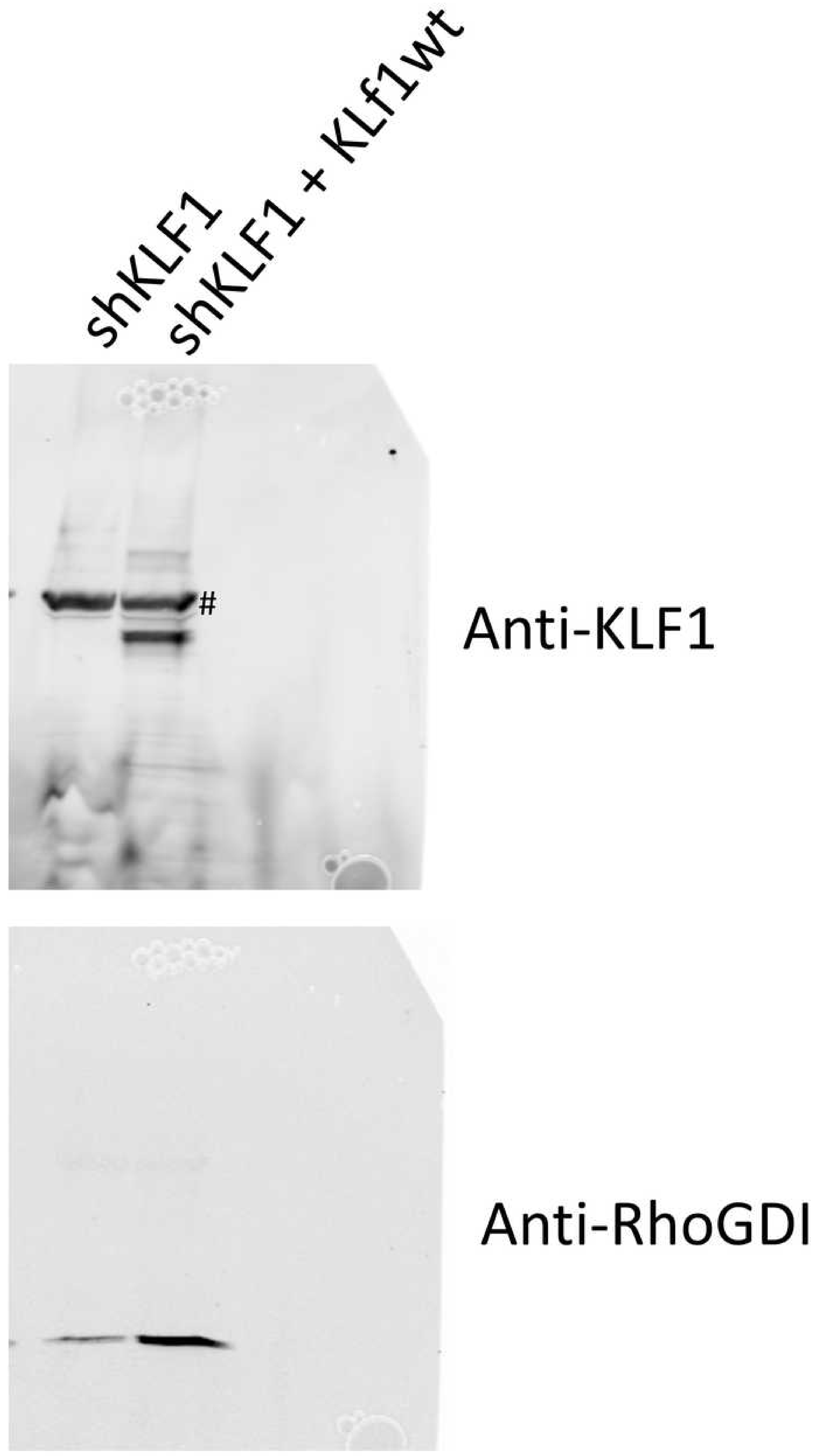

shKLF1
shKLF1 + KLf1wt
#
Anti-KLF1
Anti-RhoGDI

Novel variants in Krueppel Like Factor 1 that cause Persistence of Fetal Hemoglobin in In(Lu)individuals

Jesse Eernstman[1], Barbera Veldhuisen[2], Peter Ligthart[2], Marieke von Lindern[1], C. Ellen van der Schoot[2], Emile van den Akker[1*]

S1 Table

Donor DNA Code	KLF1 mutation	Deduced protein change	KLF1 mutation class	SNP code	Predicted effect on protein	KLF1 promoter mutation (rs79334031)	dbSNP p.Ser102Pro* (rs2072597)	HbF (%)	HbA2 (%)	HbBA1 (%)	CD44 (GM)	BAND3 (GM)	CD235 (GM)	Lu A (%)	LuA cells (GM)	Lu B (%)	LuB cells (GM)	Lu negative	Lutheran MLPA	Lutheran Serology
E13-606	c.115A>C*	p.Met39Leu*	1	rs112631212			304TC	0,81	2,95	89,03	1771	30865	10472	0,87	168	7,47	342	yes	LuBB XX	Lu(a-b-)
E14-209	c.704dupT	p.Leu236Profs*117	3	NA	Zinc finger functionality loss		304TC	1,30	3,11	84,75	705	43226	9407	0,41	117	0,76	164	yes	LuBB XY	Lu(a-b-)
E13-897	c.813G>A	p.Trp271X	3	NA	Loss of 3 zinc fingers		304TT	5,02	3,31	85,42	1638	22060	10267	1,88	160	1,47	229	yes	LuBB XX	Lu(a-b-)
E13-833	c.862A>G*	p.Lys288Glu*	2	NA			304CC	1,76	3,58	85,47	1522	72711	29714	1,00	190	2,92	251	yes	LuBB XX	Lu(a-b-)
E13-859	c.917A>T	p.Glu306Val	2	NA			304TC	0,99	2,11	89,77	3279	34457	11379	0,51	112	8,72	399	yes	LuBB XY	Lua- Lu(b)weak
E14-089	c.954G>C*	p.Trp318Cys*	2	rs769526751			304TC	0,68	2,89	89,71	1508	19117	10861	1,74	201	4,97	280	yes	LuBB XY	Lu(a-b-)
E13-677	c.977T>G*	p.Leu326Arg*	2	rs397514634			304TC	0,78	3,00	88,74	1428	20543	12050	0,46	43	5,14	321	yes	LuBB XX	Lu(a-b-)
E13-873	c.1001C>T	p.Thr334Met	2	NA		g.-148G>A*	304TC	1,04	2,83	88,27	1020	18933	6610	0,42	31	2,62	225	yes	LuBB XY	Lu(a-b-)
E13-886	c.1042T>C	p.Phe348Leu	2	NA			304TT	0,96	3,13	87,37	1428	51112	10798	0,70	144	3,75	238	yes	LuBB XX	Lu(a-b-)
E13-561	c.1042T>C	p.Phe348Leu	2	NA			304TC	0,99	3,15	88,42	1046	27836	13857	0,71	145	2,37	183	yes	LuBB XX	Lu(a-b-)
E14-024	c.1057C>T	p.His353Tyr	2	NA			304TT	1,36	3,31	87,44	1806	20004	10203	1,74	193	2,77	244	yes	LuBB XY	Lu(a-b-)
E13-621	c.1057C>T	p.His353Tyr	2	NA		g.-148G>A*	304TC	1,53	3,19	87,68	1565	46926	9423	1,14	198	3,69	257	yes	LuBB XX	Lu(a-b-)
E13-758	c.1060C>G*	p.Leu354Val*	2	rs1397962733		g.-148G>A*	304TC	0,88	3,37	86,78	1388	24463	7503	0,88	183	4,39	298	yes	LuBB XY	Lu(a-b-)
E13-834	c.1060C>G*	p.Leu354Val*	2	rs1397962733			304TT	1,08	2,89	88,12	1571	33254	16443	0,66	122	6,51	274	yes	LuBB XY	Lu(a-b-)
E13-813	c.1060C>G*	p.Leu354Val*	2	rs1397962733			304TT	0,65	2,80	88,31	1674	32989	16780	0,73	135	6,51	298	yes	LuBB XY	Lua- Lu(b)weak
E13-882	c.1060C>G*	p.Leu354Val*	2	rs1397962733			304TC	1,57	2,51	85,88	1633	14539	7259	11,30	406	6,07	286	yes	LuAB XX	Lu(a-b-)
E13-888	c.1060C>G*	p.Leu354Val*	2	rs1397962733			304TT	0,72	2,76	88,97	2134	26364	10565	0,81	153	10,84	405	yes	LuBB XX	Lua- Lu(b)weak
E13-915	c.1060C>G*	p.Leu354Val*	2	rs1397962733			304TT	0,77	2,56	88,76	1603	17987	6881	4,81	208	4,74	265	yes	LuBB XY	Lu(a-b-)
E13-761	c.1060C>G*	p.Leu354Val*	2	rs1397962733			304TT	0,80	2,80	88,06	1384	19359	10034	0,79	180	4,68	269	yes	LuBB XY	Lu(a-b-)
E13-814	c.1060C>G*	p.Leu354Val*	2	rs1397962733			304TT	0,62	3,37	88,99	2275	50453	18442	1,79	130	4,72	254	yes	LuBB XY	Lu(a-b-)
E13-653	c.1060C>G*	p.Leu354Val*	2	rs1397962733		g.-148G>A*	304TC	0,83	2,72	87,30	1594	25522	12991	0,74	140	5,05	286	yes	LuBB XX	Lu(a-b-)
E13-756	c.1060C>G*	p.Leu354Val*	2	rs1397962733			304TT	0,64	3,00	88,74	1569	16711	7503	0,66	119	3,76	202	yes	LuBB XX	Lu(a-b-)
E13-562	c.1060C>G*	p.Leu354Val*	2	rs1397962733			304TC	0,90	2,96	90,08	1596	27403	13049	0,64	131	7,71	294	yes	LuBB XX	Lu(a-b-)
E13-546	c.1060C>G*	p.Leu354Val*	2	rs1397962733			304TT	0,92	2,47	87,74	1534	36713	15659	1,61	178	10,60	347	yes	LuBB XX***	Lu(a)weak Lub-***
E13-790							304TC	0,90	2,62	89,17	3306	22639	9315	1,10	186	6,06	183	yes	LuBB XY	Lu(a-b-)
E13-874							304TC	0,73	3,00	88,09	2923	19174	6624	10,37	1	6,50	227	yes	LuAB XY	Lu(a-b-)
E13-849							304TC	0,71	2,80	88,00	3098	21468	11652	0,83	157	8,97	344	yes	LuBB XX	Lu(a-b-)
E13-652							304TC	0,91	2,69	87,41	2142	40754	9634	0,89	134	2,63	142	yes	LuBB XX	Lu(a-b-)
E13-679							304TC	0,64	3,06	87,22	1364	18351	11093	1,39	204	4,91	307	yes	LuAB XX	Lua+ Lu(b)weak
E14-110							304TC	0,87	2,42	88,07	2300	25431	11616	1,00	177	3,80	250	yes	LuBB XX	Lu(a-b-)
E13-898	c.1003G>A*	p.Gly335Arg*	2	rs750987930			304TC	1,01	2,98	86,79	1890	24684	13090	19,00	162	5,16	271	no	LuBB XX	Lu(a-b-)
E14-015	c.1060C>G*	p.Leu354Val*	2	rs1397962733			304TT	0,59	3,05	90,53	2993	30130	9517	0,68	53,3	19,75	730	no	LuBB XY	Lu(a-b-)
E13-544							304CC	0,76	2,51	88,60	2609	31330	11844	17,20	396	8,27	308	no	LuAB XX	Lu(a)weak Lub-
E14-088							304TT	0,93	2,84	90,10	3297	7952	26361	16,60	426	11,01	350	no	LuAB XY	Lu(a-b-)
E13-545							304CC	0,53	2,66	90,90	2273	51767	13655	14,73	386	11,23	297	no	LuAB XX	Lu(a)weak Lub-
E14-055							304TT	0,85	2,73	88,81	3452	23106	11255	15,15	406	11,54	353	no	LuAB XX	Lu(a)weak Lu(b)weak
E13-659							304TC	1,02	2,18	87,93	2222	25650	10335	24,50	499	12,00	386	no	LuBB XX***	Lu(a)weak Lu(b)weak***
E13-848							304TT	0,76	2,57	88,80	2342	25067	9795	1,00	192	12,00	387	no	LuAB XX	Lu(a)weak Lu(b)weak
E13-860							304TC	0,75	2,61	88,80	2390	18831	8821	29,00	574	12,02	358	no	LuAB XY	Lu(a)weak Lu(b)weak
E13-598							304TT	0,59	2,52	90,99	4580	40816	13188	41,05	706	12,25	418	no	LuAB XY	Lua+ Lub-
E14-053							304TT	0,55	2,88	89,95	3722	18629	10837	46,20	985	12,70	384	no	LuAB XY	Lua+ Lub-
E13-856							304TC	0,78	2,52	90,16	2786	19855	9962	21,30	412	12,80	384	no	LuAB XX	Lu(a-b-)
E13-852							304TT	0,75	2,35	87,87	3190	22626	7690	28,30	491	13,20	388	no	LuAB XX	Lu(a)weak Lu(b)weak
E13-619							304TC	0,62	3,05	91,68	2642	70157	17779	31,15	629	14,25	393	no	LuAB XY	Lua+ Lub-
E13-734							304TC	0,63	2,57	88,65	2463	16368	6093	0,86	179	17,30	505	no	LuBB XY	Lua- Lu(b)weak
E13-748							304TT	0,57	2,67	88,78	2603	32034	12798	0,76	153	18,00	428	no	LuBB XY	Lua- Lu(b)weak
E13-832**							304CC	0,56	3.85**	48.12**	2634	24770	7201	1,50	201	21,95	568	no	LuBB XY	Lua- Lu(b)weak
E14-067							304TT	0,72	2,64	89,51	4353	28563	9329	1,19	170	34,70	737	no	LuBB XX***	Lua+ Lu(b)weak***
E13-607							304TT	0,79	1,89	88,76	1972	31396	7489	34,80	651	11,01	322	no	LuAB XX	Lu(a)weak Lub-
E13-720							304TC	0,67	2,80	89,57	3200	13795	7808	60,55	1526	1,65	183	no	LuAB XY	Lua+ Lub-
E13-543							304TT	0,89	2,71	89,40	3536	30241	8588	42,10	955	1,67	187	no	LuAA XX	Lu(a)weak Lub-
E13-811							304TC	0,66	2,66	90,13	3472	30899	12400	37,90	794	2,83	264	no	LuAB XY	Lu(a)weak Lu(b)weak
E14-087							304TT	0,69	3,14	86,85	3520	13026	11626	35,75	795	8,56	311	no	LuAB XX	Lu(a)weak Lub-
E13-633							304TT	0,99	2,58	88,18	2132	28843	11217	45,25	919	10,17	346	no	LuAB XX	Lu(a)weak Lub-
E13-916							304TT	0,71	2,55	88,65	2051	19183	9062	23,30	493	10,30	338	no	LuAB XX	Lu(a-b-)

cell colors red, yellow and, green resemble the KLF1 mutation class. Grey/blue cells indicate Lutheran weak, negative or positive control donors.

* known mutations

** Sickle cell carrier (excluded from HbA statistics, see Figure 4C)

*** Indication when MLPA test did not match serology test

S2 Table

Primer	Sequence	Location		
Exon 1 forward	GAAGTTTGTGCCCCAGAAACAG	Promoter		
Exon 1 reverse	AAACCCCTAGACCACCCTCCTC	Intron 1		
Exon 2.1 forward	ACAGGCAAACAAGACCCCTTTC	Intron 1		
Exon 2.1 reverse	CGGGTACACCGGTTGCAG	Exon 2		
Exon 2.2 forward	GGCTCCCGACGCCTTCGT	Exon 2		
Exon 3.1 reverse	AGAGGCCAGCCAAGTCCAAGTC	Intron 2		
Exon 3.2 forward	CCAGCCCAGGCTGAGTAAAGG	Intron 2		
Exon 3.2 reverse	GCTGTCTATGGGTCCGTGTTTG	Exon 3		
Promoter 1 forward	TGGCTTTGGACACAGGGTTAGTCT	Promoter		
Pomoter 1 reverse	TCAGTGTGCTGATGGAGGGCAA	Exon 1		
Promoter 2 forward	CCAGGCTAATTTGAAGACCC	Promoter		
Pomoter 2 reverse	GGCGGGACTTGGCACGAGC	Promoter		

S3 Table

**Flow Cytometry antibodies (Human)**				
**Target of staining**	**Secundairy staining**	**Clone/Ref**	**Dilution**	
cd235	none	lot 20007845, Dako	1:200	
cd71	none	lot 5130819296, MACS miltenyi biotec	1:200	
Lu(A)	secundairy goat anti human apc	Lu A (pelikluster)	1:10	
Lu(B)	secundairy goat anti human apc	Lu B (pelikluster)	1:10	
Lutheran (total)	secundairy goat anti mouse pe	Bric224, anti-mouse IgG1, Sanquin	1:50	
CD44	none	lot 220261, BD Pharmingen	1:200	
Band 3	secundairy goat anti mouse pe	Bric6, anti-mouse IgG, Sanquin	1:10	
Mouse IgG1 APC	none	lot 3130519, GaM, BDP	1:200	
Mouse IgG APC	none	lot 28334, RaM, BDP	1:200	
PE isotype	none	lot 03428, IgG, BDP	1:200	
APC isotype	none	lot E10294-1637, IgG, eBioscience	1:200	
2ND Ab APC control	none	Lot E07254-1631, RaM, eBioscience	1:200	
**Western Blot antibodies (Human)**				
**Antibody specificity**	**Visualization**	**Clone/Ref**	**Dilution**	
KLF1 (goat)	-	ab2483, Abcam	1:1000	
2nd ab poly. rabbit anti-goat	HRP	p0449, Dako	1:5000	
V5 tag (mouse or goat)	-	KT90, Abnova	1:1000	
MAB mouse RhoGDI2	-	16, Abnova	1:1000	
-				
-				

S4 Table

Construct	Primer sequence	Sense
c.704dupT loss of zinc fingers 235	CGTCCCCCTCCTTTCCTGAGTTGTTTGGG	fw
	CCCAAACAACTCAGGAAAGGAGGGGGACG	rv
c.1042T>C, Leucine>Phenyl-alanine p.348	GCTCTGCCCACGTGCTCTTTCGCGCTCTG	fw
	CAGAGCGCGAAAGAGCACGTGGGCAGAGC	rv
Thryptophan>stop p.271, c.813G>A	CGACGTTCGTGAGCGCGCAAGAGGCAGGC	fw
	GCCTGCCTCTTGCGCGCTCACGAACGTCG	rv

S5 Table				
Figure 3 A. One-way ANOVA. CD44				
Tukey's multiple comparisons test	Mean Diff.	95% CI of diff.	Significant?	Summary
Normal (control) vs. Lu weak	129.5	-537.5 to 796.5	No	ns
Normal (control) vs. Lu negative	539.5	-475.9 to 1555	No	ns
Normal (control) vs. In(Lu)	1450	789.9 to 2111	Yes	****
Lu weak vs. Lu negative	410.1	-590.0 to 1410	No	ns
Lu weak vs. In(Lu)	1321	684.4 to 1957	Yes	****
Lu negative vs. In(Lu)	910.9	-84.86 to 1907	No	ns
Figure 4 A. One-way ANOVA. HbA				
Tukey's multiple comparisons test	Mean Diff.	95% CI of diff.	Significant?	Summary
Control donors vs. Lu weak	-0.7911	-2.909 to 1.326	No	ns
Control donors vs. Lu negative	-0.2803	-2.516 to 1.955	No	ns
Control donors vs. In(Lu)	0.4483	-1.631 to 2.528	No	ns
Lu weak vs. Lu negative	0.5108	-0.8285 to 1.850	No	ns
Lu weak vs. In(Lu)	1.239	0.1807 to 2.298	Yes	*
Lu negative vs. In(Lu)	0.7287	-0.5494 to 2.007	No	ns
Figure 4 B. One-way ANOVA. HbA2				
Tukey's multiple comparisons test	Mean Diff.	95% CI of diff.	Significant?	Summary
Control donors vs. Lu weak	-0.2181	-0.5984 to 0.1621	No	ns
Control donors vs. Lu negative	-0.2742	-0.7159 to 0.1675	No	ns
Control donors vs. In(Lu)	-0.4774	-0.8485 to -0.1063	Yes	**
Lu weak vs. Lu negative	-0.05606	-0.4209 to 0.3088	No	ns
Lu weak vs. In(Lu)	-0.2593	-0.5344 to 0.01594	No	ns
Lu negative vs. In(Lu)	-0.2032	-0.5585 to 0.1521	No	ns
Figure 4 C. Lu negative vs. In(Lu)	In(Lu)			
vs	vs			
Column A	Control donors			
Unpaired t test with Welch's correction				
P value	0.0134			
P value summary	*			
Significantly different? (P < 0.05)	Yes			
One- or two-tailed P value?	Two-tailed			
Welch-corrected t, df	t=2.642 df=27.66			
How big is the difference?				
Mean ± SEM of column A	0.6463 ± 0.06333 N=8			
Mean ± SEM of column E	1.150 ± 0.1798 N=24			
Difference between means	0.5038 ± 0.1906			
95% confidence interval	0.1130 to 0.8945			
R square	0.2016			
F test to compare variances				
F, DFn, Dfd	24.19, 23, 7			
P value	0.0002			
P value summary	***			
Significantly different? (P < 0.05)	Yes			
**Figure 6 E Column analysis. T test, paired. CD44. 72hours**				
Table Analyzed	CD44 72h post mutant transduction			
Column D	SH + WT			
vs.	vs.			
Column C	SH			
Paired t test				
P value	0.0339			
P value summary	*			
Significantly different? (P < 0.10)	Yes			
One- or two-tailed P value?	Two-tailed			
t, df	t=5,292 df=2			
Number of pairs	3			
Table Analyzed	CD44 72h post mutant transduction			
Column F	SH + 813G>C			
vs.	vs.			
Column D	SH + WT			
Paired t test				
P value	0.0377			
P value summary	*			
Significantly different? (P < 0.05)	Yes			
One- or two-tailed P value?	Two-tailed			
t, df	t=5,001 df=2			
Number of pairs	3			
Table Analyzed	CD44 log 72h post mutant transduction			
Column G	SH + 704dupT			
vs.	vs.			
Column D	SH + WT			
Paired t test				
P value	0.0308			
P value summary	*			
Significantly different? (P < 0.05)	Yes			
One- or two-tailed P value?	Two-tailed			
t, df	t=5,565 df=2			
Number of pairs	3			
Table Analyzed	CD44 log 72h post mutant transduction			
Column D	SH + WT			
vs.	vs.			
Column B	sc-shRNA			
Paired t test				
P value	0.1971			
P value summary	ns			
Significantly different? (P < 0.05)	No			
One- or two-tailed P value?	Two-tailed			
t, df	t=1,905 df=2			
Number of pairs	3			
Table Analyzed	CD44 log 72h post mutant transduction			
Column D	SH + WT			
vs.	vs.			
Column A	Untreated			
Paired t test				
P value	0.005			
P value summary	**			
Significantly different? (P < 0.05)	Yes			
One- or two-tailed P value?	Two-tailed			
t, df	t=14,13 df=2			
Number of pairs	3			
**Figure 6 F Column analysis. T test, paired. BCAM. 72hours**				
Table Analyzed	BCAM log 72h post mutant transduction			
Column D	SH + WT			
vs	vs			
Column C	SH			
Paired t test				
P value	0.1018			
P value summary	ns			
Significantly different? (P < 0.05)	No			
One- or two-tailed P value?	Two-tailed			
t, df	t=2,890 df=2			
Number of pairs	3			
Table Analyzed	BCAM 72h post mutant transduction			
Column E	SH + 1042T>C			
vs.	vs.			
Column D	SH + WT			
Paired t test				
P value	0.1498			
P value summary	ns			
Significantly different? (P < 0.05)	No			
One- or two-tailed P value?	Two-tailed			
t, df	t=2,284 df=2			
Number of pairs	3			
Table Analyzed	BCAM 72h post mutant transduction			
Column F	SH + 813G>C			
vs.	vs.			
Column D	SH + WT			
Paired t test				
P value	0.1182			
P value summary	ns			
Significantly different? (P < 0.05)	No			
One- or two-tailed P value?	Two-tailed			
t, df	t=2,645 df=2			
Number of pairs	3			
Table Analyzed	BCAM 72h post mutant transduction			
Column G	SH + 704dupT			
vs.	vs.			
Column D	SH + WT			
Paired t test				
P value	0.1156			
P value summary	ns			
Significantly different? (P < 0.05)	No			
One- or two-tailed P value?	Two-tailed			
t, df	t=2,679 df=2			
Number of pairs	3			
Table Analyzed	BCAM 72h post mutant transduction			
Column D	SH + WT			
vs	vs			
Column A	Untreated			
Paired t test				
P value	0.0776			
P value summary	ns			
Significantly different? (P < 0.05)	No			
One- or two-tailed P value?	Two-tailed			
t, df	t=3,378 df=2			
Number of pairs	3			
Table Analyzed	BCAM 72h post mutant transduction			
Column D	SH + WT			
vs.	vs.			
Column B	sc-shRNA			
Paired t test				
P value	0.0937			
P value summary	ns			
Significantly different? (P < 0.05)	No			
One- or two-tailed P value?	Two-tailed			
t, df	t=3,033 df=2			
Number of pairs	3			
Figure 7 B One-way ANOVA. HbF. 72hours				
Tukey's multiple comparisons test	Mean Diff.	95% CI of diff.	Significant?	Summary
Untreated vs. sc-shRNA	-255.5	-2969 to 2458	No	ns
Untreated vs. SH	-1903	-4616 to 811.2	No	ns
Untreated vs. SH + WT	-1237	-3950 to 1477	No	ns
Untreated vs. SH + 1042T>C	-1900	-4613 to 814.2	No	ns
Untreated vs. SH + 813G>C	-3104	-5818 to -390.3	Yes	ns
Untreated vs. SH + 703_704insT	-4498	-7211 to -1784	Yes	**
sc-shRNA vs. SH	-1647	-4361 to 1067	No	ns
sc-shRNA vs. SH + WT	-981	-3695 to 1733	No	ns
sc-shRNA vs. SH + 1042T>C	-1644	-4358 to 1070	No	ns
sc-shRNA vs. SH + 813G>C	-2849	-5562 to -134.8	Yes	ns
sc-shRNA vs. SH + 703_704insT	-4242	-6956 to -1528	Yes	*
SH vs. SH + WT	666	-2048 to 3380	No	ns
SH vs. SH + 1042T>C	3	-2711 to 2717	No	ns
SH vs. SH + 813G>C	-1202	-3915 to 1512	No	ns
SH vs. SH + 703_704insT	-2595	-5309 to 118.7	No	ns
SH + WT vs. SH + 1042T>C	-663	-3377 to 2051	No	ns
SH + WT vs. SH + 813G>C	-1868	-4581 to 846.2	No	ns
SH + WT vs. SH + 703_704insT	-3261	-5975 to -547.3	Yes	*
SH + 1042T>C vs. SH + 813G>C	-1205	-3918 to 1509	No	ns
SH + 1042T>C vs. SH + 703_704insT	-2598	-5312 to 115.7	No	ns
SH + 813G>C vs. SH + 703_704insT	-1394	-4107 to 1320	No	ns
Supplemental Figure 1 D. One-way ANOVA. Lutheran				
Tukey's multiple comparisons test	Mean Diff.	95% CI of diff.	Significant?	Summary
High vs. Medium	0.3317	-18.06 to 18.72	No	ns
High vs. Low	5.523	-10.93 to 21.97	No	ns
Medium vs. Low	5.192	-13.20 to 23.58	No	ns
Supplemental Figure 1 E One-way ANOVA. Lutheran				
Tukey's multiple comparisons test	Mean Diff.	95% CI of diff.	Significant?	Summary
High vs. Medium	11.23	3.536 to 18.93	Yes	*
High vs. Low	11,17	4.282 to 18.05	Yes	**
Medium vs. Low	-0.06667	-7.764 to 7.630	No	ns
Supplemental Figure 1 F One-way ANOVA. Lutheran				
Tukey's multiple comparisons test	Mean Diff.	95% CI of diff.	Significant?	Summary
High vs. Medium	5.943	3.461 to 8.426	Yes	***
High vs. Low	5,593	3.111 to 8.076	Yes	**
Medium vs. Low	-0.35	-2.833 to 2.133	No	ns
Supplemental Figure 1 G One-way ANOVA. Lutheran				
Tukey's multiple comparisons test	Mean Diff.	95% CI of diff.	Significant?	Summary
High vs. Medium	6.037	-0.07140 to 12.14	No	ns
High vs. Low	6,507	1.043 to 11.97	Yes	*
Medium vs. Low	0.47	-5.638 to 6.578	No	ns
Supplemental Figure 6 G One-way ANOVA. rs2072597 phenotype				
Tukey's multiple comparisons test	Mean Diff.	95% CI of diff.	Significant?	Summary
304CC vs. 304TC	6.351	-15.03 to 27.74	No	ns
304CC vs. 304TT	-2,567	-24.01 to 18.88	No	ns
304TC vs. 304TT	-8.918	-20.07 to 2.235	No	ns
Supplemental Figure X. One-way ANOVA. BAND3				
Normal (control) vs. Lu weak	-3801	-22451 to 14849	No	ns
Normal (control) vs. Lu negative	-1267	-23490 to 20955	No	ns
Normal (control) vs. In(Lu)	-7196	-25788 to 11397	No	ns
Lu weak vs. Lu negative	2534	-13248 to 18315	No	ns
Lu weak vs. In(Lu)	-3395	-13440 to 6651	No	ns
Lu negative vs. In(Lu)	-5928	-21642 to 9785	No	ns
Supplemental Figure 3 X. One-way ANOVA. Glycophorin A				
Normal (control) vs. Lu weak	2151	-3420 to 7722	No	ns
Normal (control) vs. Lu negative	3255	-3926 to 10435	No	ns
Normal (control) vs. In(Lu)	1237	-4307 to 6781	No	ns
Lu weak vs. Lu negative	1104	-4813 to 7020	No	ns
Lu weak vs. In(Lu)	-913.5	-4679 to 2852	No	ns
Lu negative vs. In(Lu)	-2017	-7908 to 3874	No	ns
